# Improved Purification and Quantitative Kinetics Monitoring of *Triatoma virus* Infection of *Rhodnius prolixus*

**DOI:** 10.3390/v18090962

**Published:** 2026-09-02

**Authors:** Everardo Gutiérrez-Millán, Gerardo Aníbal Marti, Dayana Nicté Vergara-Ortega, Mario H. Rodríguez

**Affiliations:** 1 Centre for Research in Infectious Diseases, National Institute of Public Health, Cuernavaca 62100, Mexico; ever.gmillan@gmail.com (E.G.-M.); nicte.vergara@gmail.com (D.N.V.-O.); 2 Centro de Estudios Parasitológicos y de Vectores (CEPAVE), CCT-La Plata, Consejo Nacional de Investigaciones Científicas y Técnicas (CONICET), Universidad Nacional de La Plata (UNLP), La Plata 1900, Argentina; gmarti@cepave.edu.ar

**Keywords:** *Triatoma virus*, *Rhodnius prolixus*, biological control, Chagas disease

## Abstract

Chagas disease control relies on chemical interventions, currently compromised by insecticide resistance. *Triatoma virus* (TrV) emerges as a biological candidate, requiring standardized methodologies to purify, quantify, and relate viral doses to biological effects in triatomines. We established a comprehensive protocol for the quantitative study of TrV using *Rhodnius prolixus* as a model, combining physical purification via sucrose gradients and absolute quantification by qPCR targeting the *TrVgp1* gene to link viral load with host survival. Primers were validated, and a plasmid standard curve was constructed for absolute quantification. Four isolation methods from *Triatoma infestans* macerates were compared, including an optimized discontinuous gradient protocol (EGM). High (6 × $10^{6}$ viral genome copies/µL) and low (2.47 × $10^{5}$ viral genome copies/µL) doses of EGM-purified TrV were evaluated in oral infections of *R. prolixus* over 16 days post-inoculation (dpi). The EGM protocol achieved the highest yields, around $10^{9}$ virus genome copies/µL of purified viral stock. Intestinal viral replication was dose-dependent, exhibiting distinct kinetic phases between low (3–7 dpi) and high (9–12 dpi) inoculum cohorts. Only the high dose significantly reduced host survival. In conclusion, this protocol allows high-yield TrV purification and absolute quantification in *R. prolixus*, establishing a robust dose–response foundation to assess triatomine susceptibility for integrated biological control programs.

## 1. Introduction

Chagas disease, caused by *Trypanosoma cruzi*, is a neglected vector-borne parasitosis in the Americas and a challenge for public health [1]. Within a complex epidemiological scenario, among the reduviid Chagas disease vectors, *Rhodnius prolixus* is a high-priority control target due to its extraordinary efficiency in colonizing human dwellings in regions of Mexico and Central America, where it maintains robust transmission cycles [2,3,4]. The control of these vectors relies on the spraying of chemical insecticides (mainly pyrethroids). However, this strategy faces a profound crisis due to the emergence of widespread genetic resistance, which raises demands for urgent new strategies [5,6,7].

*Triatoma virus* (TrV) is a promising candidate for integrated Chagas disease biological control [8,9]. A member of the *Dicistroviridae* family, TrV is so far the only entomopathogenic virus identified in triatomines; its non-enveloped particles measure approximately 30 nm in diameter as observed by electron microscopy in purified preparations. The viral genome consists of a single molecule of single-stranded RNA with an estimated molecular weight of 3 × 10^6^. The capsid is composed of roughly 35% RNA and 65% protein by weight, containing three major structural polypeptides of 39 kDa, 37 kDa, and 33 kDa, along with a minor component of approximately 45 kDa—features consistent with its classification as a small picorna-like virus [10,11,12,13]. Previous studies showed that it exhibits strict taxonomic host specificity, with a narrow restriction to the Triatominae subfamily [14,15]. Its potential as a biological control agent lies in its biological aggressiveness: it replicates in the insect’s intestinal epithelium, causing a high mortality rate, reduced fecundity, and delayed moulting processes [13,16,17,18]. In addition, its horizontal transmission via coprophagy (ingestion of infected feces) aligns perfectly with the gregarious behaviour of the vectors, allowing effective dispersal within breeding and hiding sites [11,15,19].

Before a rigorous validation of the biosafety and ecological security of TrV-based interventions, documentation of the virus infectivity and standardization of the methodology to determine parameters such as inoculum size and measurement of virus loads in infected bugs are necessary.

Currently, physical purification methods for TrV are inefficient; thus, resolution and quantitative viral load curves to measure the replication of the virus over time or link it to its pathogenic impact in a reproducible manner are inaccurate. Also, the absence of homologous cell lines impedes classic in vitro titration assays and standardized replication studies [9,20]. Consequently, most diagnoses are limited to qualitative RT-PCR, AC-ELISA, or electron microscopy techniques, which offer binary responses (presence/absence) but prevent standardization of precise doses [8,21,22,23].

The objective of this work was to establish an integral protocol as a new standard for the quantitative study of TrV infection using *R. prolixus* as a model. Our methodological proposal combines physical purification resolved by sucrose density gradients with absolute quantification by qPCR targeting the *TrVgp1* gene. This method allows for a direct and precise link between the viral load and the survival of the host. To demonstrate the protocol’s sensitivity, we evaluated the infection dynamics by comparing a high versus a low dose over 16 days, providing a fundamental analytical tool for the future of Chagas disease biocontrol.

## 2. Materials and Methods

### 2.1. Rhodnius prolixus as a Study Model

Adult *R. prolixus* specimens maintained at the insectary of the Instituto Nacional de Salud Pública were used. The insects were kept inside an incubator at a constant temperature of 28 °C and a controlled relative humidity of 60–70%. The adults were fed rabbit blood via artificial feeders every 15 days.

### 2.2. Triatoma virus TrVgp1 and TrVgp2 Primer Design

The Primer-BLAST tool v2.17 [24] was used to design two primer pairs using the TrV reference genome (NCBI Accession ID: NC_003783.1) targeting two distinct viral regions:

- *TrVgp1* (Forward: 5′-CAGACGGCTATGAGTGGTATTGA-3′, Reverse: 5′-TCCACAGCCAAAGGAGTATCAAA-3′, 164 bp) was directed at a site near the RNA-dependent RNA polymerase (RdRp) domain of the Dicistroviridae.
- *TrVgp2* (Forward: 5′-TCTGTTGTTTCTGCTCCTGTTCT-3′, Reverse: 5′-GCAGAATGACCAAGCAGAATGTT-3′, 113 bp) targeted the Cricket paralysis virus (CrPV) capsid protein region. The *TrVgp1* primer pair was utilized for all subsequent viral quantification assays.

### 2.3. Total RNA Extraction and cDNA Synthesis from Macerated Triatoma infestans Samples

TrV inoculum was obtained from a chronically infected laboratory colony of *T. infestans* maintained under controlled biosecurity conditions at the Centro de Estudios Parasitológicos y de Vectores (CEPAVE; CONICET–Universidad Nacional de La Plata, Argentina). The viral source consisted of naturally deceased adult insects of both sexes exhibiting characteristic TrV-induced abdominal distension. To prepare the viral stock, heads, legs, and thoraces were removed, and the remaining abdomens were desiccated in a laboratory oven at 40–50 °C for 24 h. Dried abdomens were homogenized into a fine powder using a mechanical grinder and systematically combined into a cumulative reference stock to ensure long-term biological homogeneity and minimize batch-to-batch variation. Approximately 2 to 3 adult abdomens yielded 0.05 g of dry tissue powder, serving strictly as a standardized physical mass starting material per extraction aliquot. From each aliquot, viral isolation and purification were performed, and absolute viral copy numbers were subsequently determined by qPCR from the reconstituted purified viral stock in TE buffer prior to experimental use. This infectious powder was stored at 4 °C until suspension in physiological buffer for viral purification.

For total RNA extraction, 0.05 g of macerated *T. infestans* abdomens was mixed with 200 µL of NMT buffer (0.01 M NaCl, 0.001 M MgCl_2_, 0.04 M Tris-HCl, pH 7.4). The mixture underwent three successive centrifugations at 15,000× *g* for 10 min at 25 °C, recovering the supernatant after each round, and 600 µL of TRIzol reagent (Thermo Fisher Scientific, Waltham, MA, USA) was added, homogenized by vortexing for 5 s, and incubated for 5 min at 25 °C. Chloroform (Sigma-Aldrich, St. Louis, MO, USA) was added at a ratio of 0.2 mL per 1 mL of TRIzol. The mixture was vortexed for 15 s, incubated for 3 min at 25 °C, and centrifuged at 20,000 *g* for 15 min at 4 °C. The upper aqueous phase was collected and treated with one-tenth of its volume of sodium acetate (3 M, pH 5.2), followed by 2.5 volumes of absolute ethanol (Sigma-Aldrich, St. Louis, MO, USA). The mixture was homogenized and incubated at −20 °C for 1 h. Following incubation, the samples were centrifuged at 20,000 *g* for 15 min at 4 °C. After discarding the supernatant, the resulting RNA pellet was air-dried. Two consecutive washes were performed with 800 µL of 75% ethanol, allowing the tubes to dry completely at each step. Finally, the precipitate was suspended in diethyl pyrocarbonate (DEPC)-treated water (Sigma-Aldrich, St. Louis, MO, USA).

RNA quantification was performed using a NanoDrop 1000 v.3.7 spectrophotometer (Thermo Fisher Scientific, Waltham, MA, USA). Complementary DNA (cDNA) synthesis was performed using an oligo(dT) primer (Thermo Fisher Scientific, Waltham, MA, USA) and the RevertAid First Strand cDNA Synthesis Kit (Thermo Fisher Scientific, Waltham, MA, USA) according to the manufacturer’s instructions. The manufacturer’s protocol was modified by proportionally halving all component volumes to achieve a final reaction volume of 13 µL. The total input of total RNA from each macerated sample was standardized to 300 ng per reaction. The synthesized cDNA preparations were stored at −70 °C until subsequent use.

### 2.4. PCR Validation and Sanger Sequencing of TrVgp1 and TrVgp2

To detect the presence of TrV, RNA was extracted from macerated *T. infestans* samples and used as a template for cDNA synthesis. Subsequently, end-point PCR was performed to identify the *TrVgp1* and *TrVgp2* regions, followed by the visualization of the amplified fragments on a 2% (*w*/*v*) agarose gel for confirmation. For both primer pairs, the PCR thermal profile consisted of initial denaturation at 95 °C for 10 min. Forty cycles of 95 °C for 30 s, 58 °C for 30 s, and 72 °C for 30 s. A final extension at 74 °C for 10 min. The resulting amplicons for *TrVgp1* and *TrVgp2* were purified and submitted for Sanger sequencing at the Instituto de Biotecnología, UNAM, Mexico. Manual sequence trimming and quality cleaning were performed using SnapGene software v8.0.1 (www.snapgene.com). Sequence alignment and consensus sequence generation were conducted via Jalview v1.8.3 [25]. Finally, the BLASTn tool v2.17 [26] was employed to query the consensus sequence against the non-redundant nucleotide (nt) database of the National Center for Biotechnology Information (NCBI) (https://www.ncbi.nlm.nih.gov).

### 2.5. Preparation of a Plasmid Standard Curve

To determine absolute viral copy numbers via qPCR, a standard curve was constructed using a recombinant pUCIDT-ampicillin plasmid containing the target sequence (TrV_dicer2_GFP-403), with a total length of 3155 bp and a molecular weight of 1,949,464.3 Da. The lyophilized plasmid (4 µg) was reconstituted in 100 µL of TE buffer (Tris–EDTA) (1 mM EDTA, 10 mM Tris–HCl, pH 7.4) to prepare a working stock solution with a final concentration of 40 ng/µL. The absolute plasmid copy number within the stock was mathematically determined using Avogadro’s number according to the following formula:

$$\mathrm{Mass}\;\mathrm{of}\;\sin\mathrm{gle}\;\mathrm{plasmid}\;\mathrm{molecule}\;\left(g\right)=\frac{\mathrm{Molecular}\;\mathrm{Weight}\;(g/\mathrm{mol})}{6.022\times \;10^{23}\;molecules/mol}=3.237\times 10^{-18}\;g$$

$$\mathrm{Total}\;\mathrm{stock}\;\mathrm{copy}\;\mathrm{number}=\frac{4\times 10^{-6}\;g}{3.237\times 10^{-18}\;g}=1.24\times 10^{12}\;copies$$

This total abundance yielded a baseline stock concentration of $1.24\times 10^{10}$ copies/µL. Subsequently, ten-fold serial dilutions were systematically prepared in TE buffer to establish a 10-point standard curve spanning concentrations from 4 ng/µL down to $4\times 10^{-7}$ ng/µL. Utilizing a template volume of 1 µL per qPCR reaction, the dynamic range of the standard curve encompassed an absolute coverage from $1.24\times 10^{9}$ down to $1.24\;\times 10^{2}$ copies/reaction.

### 2.6. qPCR to Quantify TrV TrVgp1

To quantify *TrVgp1* expression, a qPCR assay was standardized on a Rotor-Gene Q 5plex HRM Platform (QIAGEN, Hilden, Germany) in a final reaction volume of 10 µL utilizing the Maxima SYBR Green qPCR Master Mix (Thermo Fisher Scientific, Waltham, MA, USA) and 2.5 µM of each specific primer. This process utilized a MiniGene 25–500 bp plasmid from Integrated DNA Technologies (IDT, Coralville, IA, USA) carrying an ampicillin resistance marker and containing the target sequence. Amplification efficiency was evaluated via serial plasmid dilutions using the standard curve method [27]. The qPCR assays were performed using the same PCR thermal profile described above. Absolute viral copy numbers were determined using the Rotor-Gene Q Software v2.3.5 (QIAGEN Rotor-Gene Q 5plex HRM System) through direct import of the validated plasmid standard curve. Quantitative evaluations were restricted to target cycle threshold ($C_{q}$) values falling strictly within the linear dynamic range of the assay ($C_{q}$ 7.75–30.68, corresponding to $2.34\times 10^{9}$ to $2.83\times 10^{2}$ viral genome copies/μL). The complete amplification profiles and melt curve analysis reports generated by the software are provided in the Supplementary data (File Report_S1 and Table_S1 within the qPCR_Reports folder).

### 2.7. Specificity Panel and Cross-Reactivity Controls

To rigorously evaluate the analytical specificity of the TrV assay and ensure no cross-reactivity occurred with co-occurring vectors or parasites, a comprehensive panel of non-target biological controls was processed:

- Non-target Triatomine Species: Total RNA was extracted from the digestive tract of colony-reared adult *R. prolixus* and *Triatoma phyllosoma* maintained under controlled laboratory conditions, as well as wild-caught adult specimens of *T. pallidipennis* collected in the field, following the protocol described in Section 2.9.
- *Trypanosoma cruzi* Culture and Harvesting: Parasites were maintained in Liver Infusion Tryptose (LIT) medium (Sigma-Aldrich, St. Louis, MO, USA) supplemented with 10% fetal bovine serum (FBS) and 1X Penicillin-Streptomycin-Neomycin (PSN) mixture (Thermo Fisher Scientific, Waltham, MA, USA), following established formulation protocols [28]. Cultures in active exponential growth were harvested by centrifugation at 2500 rpm for 3 min at room temperature, and cell densities were quantified using a Neubauer counting chamber.
- *T. cruzi* Culture Isolates and Infected Vectors: The panel included pure epimastigote cultures of five distinct *T. cruzi* strains (Morelos, Chalcatzingo, Tc19, Valentina, and Chilpancingo). Additionally, fifth-instar (N5) nymphs of *T. pallidipennis* were experimentally infected with each of these five strains via artificial feeding at a concentration of $4\times 10^{6}\;\mathrm{epimastigotes}/\mathrm{mL}$. Tissues from infected N5 nymphs were collected and processed at 20 days post-inoculation (dpi).

All non-target insect tissues and parasite cultures were extracted following the same RNA extraction and cDNA synthesis protocols used for the experimental samples. To confirm nucleic acid integrity and the presence of amplifiable parasite cDNA in the *T. cruzi* group prior to specificity testing, all 14 samples (pure cultures and infected N5 *T. pallidipennis* tissues) were independently validated using the *T. cruzi*-specific Cyb1 qPCR system targeting the mitochondrial cytochrome b (*Cyb1*) gene (66 bp fragment, $T_{m}\approx {70\;}^{\circ }C$), with primers *Cyb1F* (5′-GAGTGGGRTTYAGTTTRGGTYT-3′) and *Cyb1R* (5′-TTCAYGCTAARCARACACCACA-3′) as described by de Oliveira et al. (2021) [29]. Subsequently, identical cDNA volumes (1.0 μL) from all control groups (*R. prolixus*, *T. phyllosoma*, wild adult *T. pallidipennis*, and *T. cruzi*-infected N5 nymphs and cultures) were subjected to both *T. cruzi* and TrV qPCR assays under standard conditions to verify the complete absence of non-specific amplification or false-positive signals in the viral assay.

### 2.8. Isolation and Purification of TrV

Four methods for the isolation and purification of TrV from *T. infestans* abdomens macerates were evaluated. All ultracentrifugation steps were performed using an Optima XPN-90 ultracentrifuge (Beckman Coulter, Brea, CA, USA). The detailed physical and kinetic parameters for each workflow are summarized in Table S1.

- Method 1, Marti [8]: The procedure replicated a multi-step ultracentrifugation procedure. Briefly, sample (0.05 g) homogenate in NMT buffer with two consecutive centrifugations at 20,000× *g* for 10 min each, to remove large cell debris. The supernatant was ultracentrifuged at 140,000× *g* for 3 h at 4 °C to pellet the viral particles. The viral pellet was suspended in NMT buffer and layered onto a continuous sucrose density gradient (10–30%, *w*/*v*), followed by ultracentrifugation at 64,000× *g* for 3 h at 4 °C. Finally, sequential 2 mL fractions were collected throughout the entire gradient and diluted in NMT buffer and ultracentrifuged at 44,000× *g* for 2 h at 4 °C to remove residual sucrose and concentrate the virus. The final purified viral pellet was resuspended in TE buffer.
- Method 2, Susevich [9]: This adapted a procedure to process single samples. Briefly, individual samples (0.05 g) were homogenized in 150 µL of phosphate-buffered saline (PBS) and subjected to three successive clarifications at 13,000 *g* for 10 min to isolate the viral particles and completely remove host debris. The final clarified supernatant was layered onto a continuous sucrose density gradient (10–30%, *w*/*v*) and ultracentrifuged at 30,000 *g* for 3 h at 4 °C. Following ultracentrifugation, consecutive fractions of 2 mL were collected from the entire gradient.
- Method 3, Optimized Discontinuous Gradient (EGM method): This protocol was developed by the authors and optimized to maximize viral recovery. Samples (0.05 g) were homogenized in 300 µL of NMT buffer. The homogenate was clarified via three successive microcentrifugations at 15,000× *g* for 10 min at 25 °C, recovering and retaining the supernatant after each round to remove large cellular debris. The final clarified supernatant was then layered onto a discontinuous sucrose density gradient of 20%, 30%, and 60% (*w*/*v*) and separated by ultracentrifugation at 82,844× *g* for 2 h at 4 °C. Following centrifugation, consecutive 2 mL fractions were collected from the entire gradient. Each individual fraction was then subjected to a second ultracentrifugation step at 44,000× *g* for 2 h at 4 °C to pellet the viral particles, and the resulting pellets were resuspended in 1 mL of TE buffer.
- Method 4, Microcentrifuge (clarification only): This method consisted exclusively of the initial separation phase to directly obtain the enriched viral supernatant, completely omitting all subsequent density gradient, washing, and ultracentrifugation steps. Samples were homogenized in 300 µL of NMT buffer. The homogenates were subjected to three successive microcentrifugations at 15,000× *g* for 10 min at 25 °C to remove host debris, and the supernatant was recovered after each step.

For total RNA extraction, 100 µL aliquots were taken from each individual gradient fraction of Method 1 and from each viral preparation obtained from Methods 2 to 4. Each aliquot was mixed with 300 µL of TRIzol reagent. From this stage forward, the procedure continued from the chloroform addition step for phase separation, followed by pellet washing and drying. cDNA was synthesized as described above using 100 ng of total RNA per reaction to ensure uniform template amounts. For the downstream qPCR analysis of the *TrVgp1* gene, a standardized template input of 100 ng of total cDNA was used per reaction.

### 2.9. Experimental Oral Infections, TrV Viral Kinetics, and Mortality in Rhodnius prolixus

Virus stocks were prepared using gradient fractions from the EGM method. To evaluate two TrV doses in independent experiments, insects were divided into groups of approximately 9 individuals. For the high-dose experiment (n=96), the viral fractions F2_1, F3_1, and F5_1 (detailed in Table S2) were pooled and adjusted to an initial stock concentration of $1.80\times 10^{7}$ viral genome copies/µL. For the low-dose experiment (n=88), the fraction F1_2 (Table S2) was diluted in TE buffer to a stock concentration of $7.41\times 10^{5}$ viral genome copies/µL. The infectious inocula were prepared by mixing the corresponding virus stock with rabbit blood in a 1:3 ratio. The final viral concentrations in the feeding mixtures were $6\times 10^{6}$ viral genome copies/µL for the high-dose group and $2.47\times 10^{5}$ viral genome copies/µL for the low-dose group. Complete qPCR run files for these inoculum determinations are available in Supplementary Data (qPCR_Reports/Reports_S2). Control groups (n=90 and n=75 for the high and low dose groups, respectively) were fed by replacing the viral stock with 200 µL of TE buffer. Blood meals were offered through an artificial membrane system equipped with glass jackets at 37 °C. The base of each feeder [30] was sealed with a tightly stretched Parafilm^®^ membrane (Bemis Company, Inc., Neenah, WI, USA) to mimic host skin. Detailed specifications regarding inoculum composition, the sampling timeline, and estimated final doses per individual are summarized in Table S3.

Following infection, all four experimental groups were maintained under controlled insectary conditions. Daily survival tracking was conducted for 16 days post-inoculation (dpi). To track viral load kinetics simultaneously within these same cohorts, insects from the high-dose group were sacrificed on days 1, 3, 5, 7, 9, 12, 14, and 16 dpi. Sampling for the low-dose group was conducted on days 1, 3, 5, 7, 9, and 12 dpi, as viral load kinetics had already reached a stationary plateau by day 12; this schedule optimization allowed conserving the remaining low-dose individuals exclusively for long-term survival monitoring because of the limited availability of the colony insects.

At each sampling dpi, the complete digestive tracts (including the stomach, intestine, and rectum) of experimental insects were dissected. To ensure that qPCR quantification reflected active viral interaction of host tissues rather than residual inoculum or viral accumulation in excretions, the dissected organs were thoroughly rinsed with PBS to completely remove blood remnants and lumen contents (feces).

The tissues were macerated using a pestle in 200 µL of TRIzol reagent; following homogenization, 200 µL of the same reagent was added. The samples were clarified at 20,000 *g* for 15 min at 25 °C in a microcentrifuge, and the supernatants were recovered. Total RNA extraction from individual insect digestive tracts was then performed, yielding total RNA that was adjusted to a standardized baseline sample of 300 ng for cDNA synthesis, and 1 µL of cDNA template was loaded per reaction for qPCR quantification of *TrVgp1*.

Mock-infected control insects (fed TE buffer in rabbit blood) were evaluated by qPCR across all sampling time points (1–16 dpi) to monitor baseline viral presence. The assay specificity was validated using melting curve analysis, establishing a target melting temperature (T_m_) threshold of 77 °C for specific *TrVgp1* amplicons and discriminating against potential non-specific artefacts or primer-dimers.

To prevent underestimating the cumulative survival probability due to the scheduled analytical sacrifices, insects removed during sampling points were mathematically integrated into Kaplan–Meier survival models [31] as right-censored data (Status = 0) at their specific time of removal.

### 2.10. Statistical Analysis

All statistical analyses and data visualizations were performed in R v4.2.1 [32] utilizing RStudio v2025.09.0+387 [33]. Data were handled using the dplyr package v1.1.4 [34], and figures were compiled using ggplot2 v3.5.2 [35] and cowplot v1.1.3 [36]. For all tests, statistical significance was set at alpha = 0.05.

Absolute viral copy numbers obtained from qPCR assays were ${log}_{10}$-transformed to fulfil normality assumptions where applicable or to reduce heteroscedasticity. To ensure data integrity, only determinations falling strictly within the linear dynamic range (LDR) of the qPCR standard curve ($2.34\times 10^{9}$ to $2.83\times 10^{2}$ viral genome copies/μL) were retained for downstream statistical analyses. This criterion was uniformly applied to both the absolute quantification of purified viral stock concentrations and tissue samples collected during infection experiments.

Differences in viral yield among the four purification fractions/methods (Marti, Susevich, EGM, and Microcentrifuge) were evaluated using the non-parametric Kruskal–Wallis test, followed by post hoc pairwise comparisons with p-value adjustments for multiple testing. Similarly, differences in viral loads between the high- and low-dose cohorts at individual time points throughout the infection kinetics (days post-inoculation) were analyzed using the Wilcoxon rank-sum test (Mann–Whitney U test).

For the mortality assays, survival curves were estimated using the non-parametric Kaplan–Meier method via the survival v3.5.5 [37] and survminer v0.5 [38] packages. Cumulative survival distributions between virus-infected insects and their respective sham-infected control groups were compared independently for each dose cohort using the Log-Rank (Mantel–Cox) test.

## 3. Results

### 3.1. Confirmation of TrVgp1 and TrVgp2 Amplicons by Sanger Sequencing

The presence of TrV in macerated *T. infestans* samples was confirmed via end-point PCR. The resulting amplification products exhibited specific bands corresponding to the *TrVgp1* and *TrVgp2* gene fragments (Figure S1). Following end-point PCR confirmation, the amplicons were sequenced. BLASTn alignment analysis of the consensus sequences revealed high nucleotide identity >98% and an E-value $<1\times 10^{-40}$ with reference *Triatoma virus* sequences deposited in GenBank, confirming that the amplified fragments correspond to this viral species (Supplementary Data: Blastn results).

### 3.2. Assay Specificity and Cross-Reactivity

To evaluate analytical specificity, the TrV qPCR assay was tested against uninfected controls (*R*. *prolixus* maintained in the insectary, external to feeding or infection experiments), non-target vector species (*T*. *phyllosoma* and wild-caught *T*. *pallidipennis*), and *T*. *cruzi* matrices.

The presence of amplifiable parasite cDNA across all 14 *T. cruzi* samples (pure epimastigote cultures of strains Morelos, Chalcatzingo, Tc19, Valentina, and Chilpancingo, as well as N5 *T. pallidipennis* nymphs at 20 dpi) was first confirmed using the parasite-specific *Cyb1* qPCR assay [29]. All *T. cruzi* culture isolates and infected nymph tissues amplified successfully with expected melting temperatures ($T_{m}=71.4\pm {0.25\;}^{\circ }C$) (Supplementary File Report S3.0) with slight $T_{m}$ shifts relative to the original report (${70.0\;}^{\circ }C$) attributable to strain-specific sequence polymorphism within the *cyb1* target fragment.

When assayed with the TrV (*TrVgp1*) system, none of the non-target triatomine species, *T. cruzi* strains, or *T. cruzi*-infected N5 nymphs produced target amplification ($C_{t}>34.0$ or undetermined) or the characteristic TrV melting peak (${77\;}^{\circ }C$) (Supplementary File Report_S3.1 and 3.2). While the positive control amplified normally ($C_{t}=29.76$, $T_{m}={77\;}^{\circ }C$), all cross-reactivity samples showed baseline noise identical to non-template controls, confirming 100% analytical specificity.

### 3.3. Comparison of TrV Purification Methods

**Figure 1:**
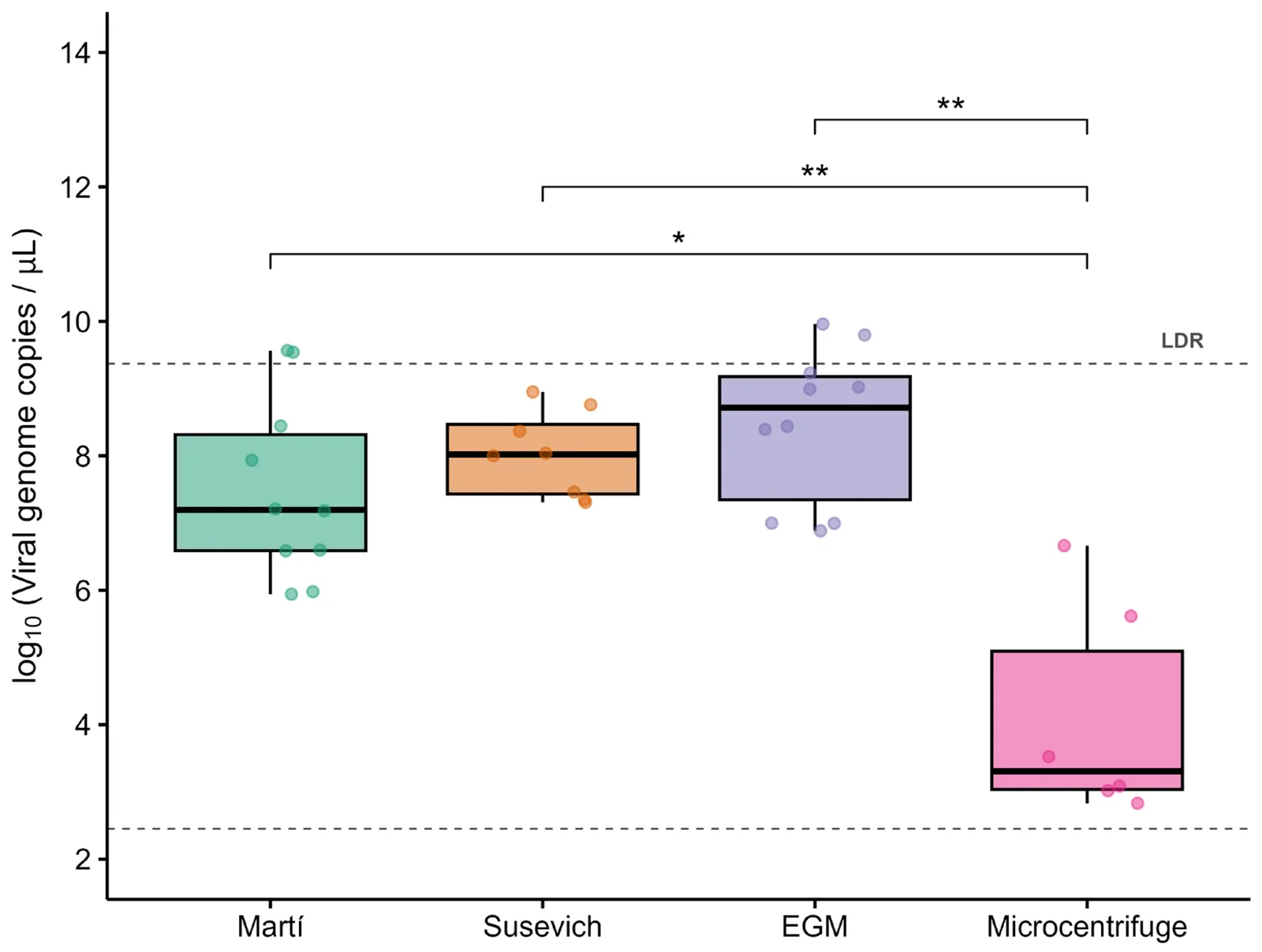
TrV viral copy yields performance of different purification methods. Viral load levels are expressed on a ${log}_{10}$ scale of total *TrVgp1* gene copies/µL determined by qPCR. Each boxplot represents an independent purification/extraction protocol: Marti (green), Susevich (orange), EGM (purple), and Microcentrifuge (pink). Individual dots correspond to the biological replicates analyzed per condition. Dashed horizontal lines indicate the upper and lower bounds of the assay’s linear dynamic range (LDR: $2.83\times 10^{2}$ to $2.34\times 10^{9}\;\mathrm{viral}\;\mathrm{genome}\;\mathrm{copies}/\mu L$). Upper brackets indicate statistically significant pairwise differences between methods (* *p* < 0.05, ** *p* < 0.01).

Quantitative evaluation via qPCR revealed significant differences in TrV recovery across the evaluated methodologies (Figure 1, Tables S2 and S4). All three density-gradient purification methods—Marti, Susevich, and the optimized EGM protocol—achieved robust viral yields within the $10^{6}$ to $10^{9}$ viral genome copies/μL range (Table S2), significantly outperforming the Microcentrifuge method (clarification only; Figure 1, Table S4). Post hoc pairwise comparisons confirmed that viral copy yields were significantly higher in Martí (p<0.05), Susevich (p<0.01), and EGM (p<0.01) protocols compared to the Microcentrifuge method (Figure 1).

Although no statistically significant differences were detected among the three density-gradient protocols themselves (p>0.05; Figure 1, Table S4), the EGM method consistently achieved the highest average viral recovery. Enriched fractions from the EGM protocol reached concentration peaks up to $9.11\times 10^{9}$ viral genome copies/μL (e.g., fraction F1_2; Table S2), combined with consistent total RNA recovery (11.5–28.5 ng/μL). The Susevich method exhibited a uniform profile with viral titers between $2\times 10^{7}$ and $8.9\times 10^{8}$ viral genome copies/μL (Table S2), while the Marti method showed high variability across fractions ($8.77\times 10^{5}$ to peaks of $3.67\times 10^{9}$ viral genome copies/μL; Table S2). Conversely, the Microcentrifuge protocol yielded the lowest viral loads ($6.78\times 10^{2}$ to $4.62\times 10^{6}$ copies/μL; Table S2; Figure 1).

### 3.4. TrV Viral Replication Kinetics and Host Survival Analysis

**Figure 2:**
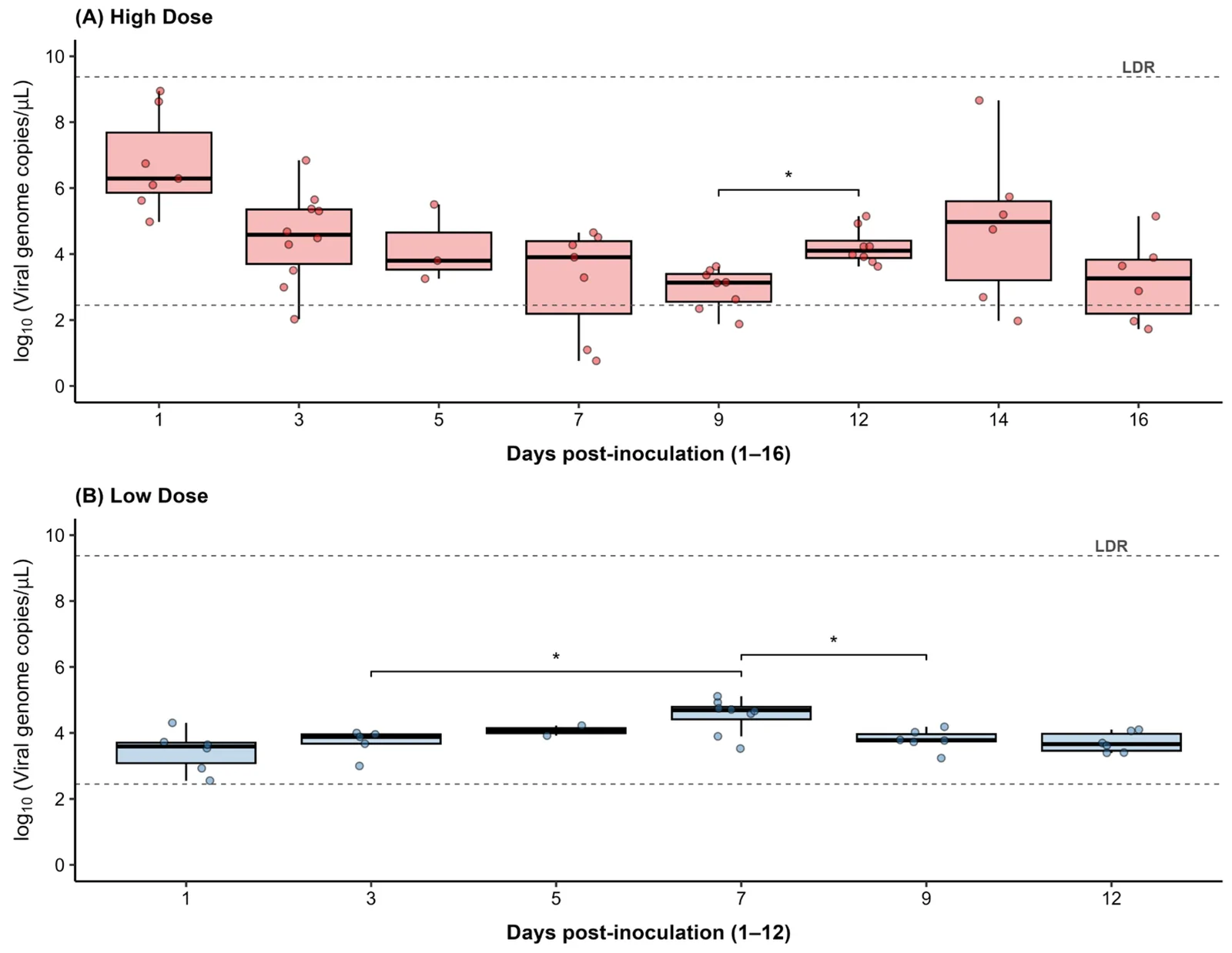
Kinetics of *Triatoma virus* (TrV) viral load in the digestive tract of *Rhodnius prolixus*. Absolute quantification of the *TrVgp1* genomic RNA expressed on a ${log}_{10}$ scale of viral genome copies/μL determined by qPCR across post-inoculation days (dpi). Each 1 μL cDNA template loaded per reaction corresponds to an equivalent input of 23.08 ng of standardized starting total RNA. (**A**) High-dose infected cohort monitored from 1 to 16 dpi. (**B**) Low-dose infected cohort monitored from 1 to 12 dpi. Horizontal lines within the boxplots represent the median, boxes indicate the interquartile range (IQR), and individual dots show biological replicates. Dashed horizontal lines define the upper and lower bounds of the assay’s linear dynamic range (LDR: $2.83\times 10^{2}$ to $2.34\times 10^{9}\;\mathrm{viral}\;\mathrm{genome}\;\mathrm{copies}/\mu L$). Brackets with asterisks denote statistically significant pairwise differences between sampling time points within the same dosage group (* p<0.05). Mock-infected control insects (fed TE buffer in rabbit blood) were verified as strictly negative for target *TrVgp1* genomic RNA via melting curve analysis (T_m_ = 77 °C) across all timepoints and are omitted from the logarithmic scale.

To evaluate the dose-dependent replication dynamics of TrV, absolute quantification targeting the *TrVgp1* gene was performed across post-inoculation days (dpi) in the digestive tract of *R. prolixus*. Analysis of the qPCR datasets confirmed that TrV infection follows a distinct, dose-dependent kinetic trajectory in *R. prolixus* (Figure 2).

**Figure 3:**
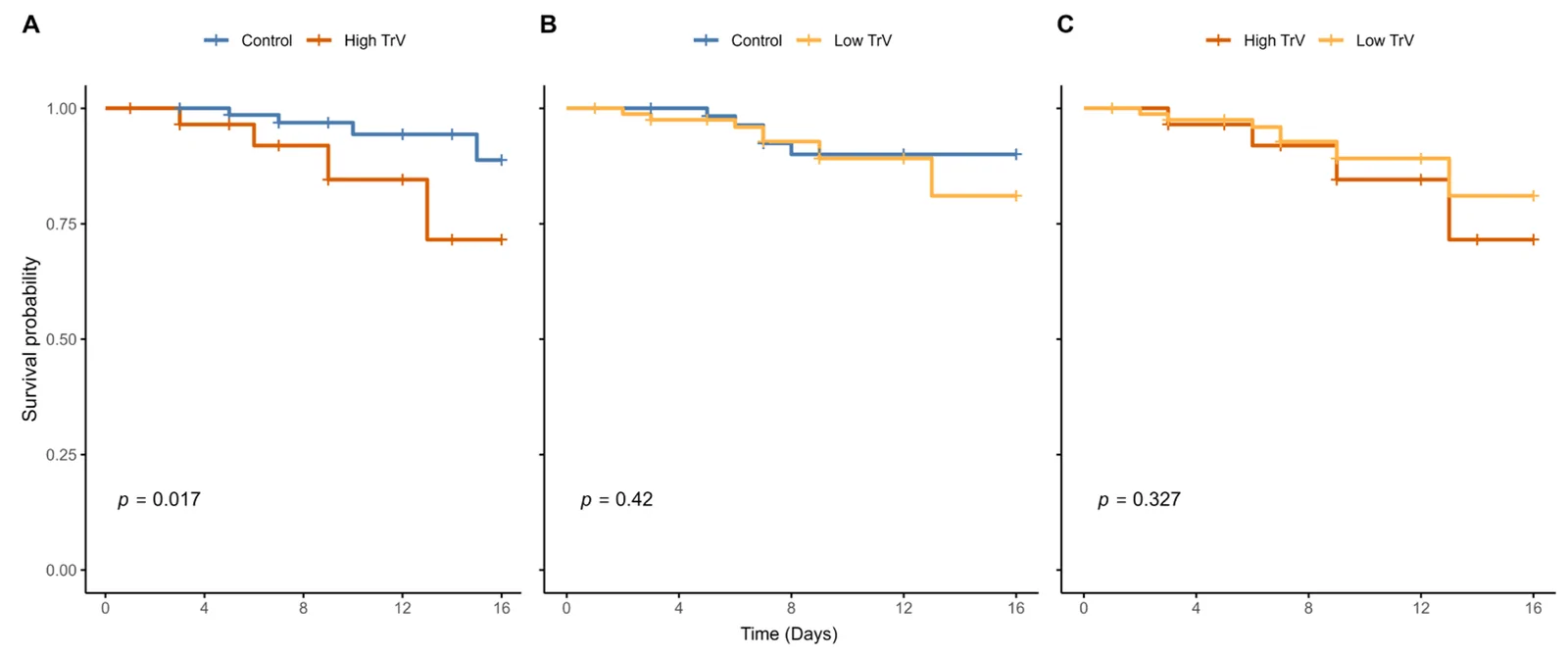
Kaplan–Meier survival curves of adult *Rhodnius prolixus* exposed to *Triatoma virus* (TrV). Cumulative survival probabilities were tracked over a 16-day post-inoculation period across experimental cohorts. (**A**) Survival curve comparison between the high-TrV-dose cohort (N=96) and uninfected control insects (N=90). (**B**) Survival curve comparison between the low-TrV-dose cohort (N=88) and uninfected control insects (N=75). (**C**) Direct pairwise dose–response survival comparison between high-TrV- and low-TrV-infected cohorts. Statistical significance between survival curves was evaluated using the Log-Rank (Mantel–Cox) test (α=0.05; nominal p-values are indicated within each panel, and summary statistics are detailed in Table S6).

In the high-dose cohort, viral loads exhibited an initial high variability at 1 dpi (mean: 6.757 ± 1.493 log_10_), representing variable ingested inoculum levels. This was followed by a sharp decline between 1 and 3 dpi. Viral loads reached an absolute nadir at 9 dpi (mean: 3.352 ± 0.219 log_10_). Crucially, a statistically significant viral surge was detected between 9 and 12 dpi (p=0.016; Table S5), with mean viral titers rising to 6.085 ± 1.764 log_10_ viral genome copies/μL and individual biological replicates reaching up to $4.57\times 10^{8}$ viral genome copies/μL at 14 dpi (Figure 2; Supplementary data: qPCR Reports/Reports_S4: Files Reports_4.0–4.2). This late viral peak coincided directly with an increase in host mortality (Figure 3, Supplementary data.xlsx: Raw mortality).

In contrast, the Low-dose cohort displayed a controlled, non-lethal kinetic profile. Starting from a modest baseline at 1 dpi (mean: 3.802 ± 0.344 log_10_), viral loads exhibited a statistically significant rise between 3 and 7 dpi (p=0.022; Table S5) (Figure 2B; Supplementary data: qPCR Reports/Reports_S5: Files Reports_5.0 and 5.1). Following this inductive phase, viral loads stabilized into a persistent kinetic plateau between 7 and 12 dpi (p=0.687; Table S5), maintaining a steady non-lethal equilibrium (mean: 3.714 ± 0.309 log_10_ at 12 dpi) without inducing host mortality across the 16-day monitoring period (0 deaths between 14 and 16 dpi; Supplementary data.xlsx: Raw mortality).

Throughout the entire monitoring timeframe (1–16 dpi), mock-infected control insects (fed TE buffer in rabbit blood) were verified as strictly negative for *TrVgp1* genomic RNA using melting curve analysis (T_m_ 77 °C). Complete amplification and melting curve reports for all control group evaluations are archived in the Supplementary Data repository (qPCR_Reports/Reports_S6 folder).

Kaplan–Meier survival analysis demonstrated that the effect of TrV on *R. prolixus* mortality was dependent on the initial viral dose (Figure 3, Table S6, Supplementary Data: Raw mortality). Exposure to the high viral dose significantly reduced insect survival probability compared to its corresponding control group (Log-Rank test, $\chi^{2}=5.66$, df = 1, p=0.0174, Figure 3A), with 14 cumulative deaths recorded in the infected cohort versus 4 in the control group by day 16. In contrast, the low viral dose did not compromise adult viability; survival curves between the infected insects (10 deaths) and their corresponding sham-infected control (5 deaths) showed no statistically significant differences (Log-Rank test, $\chi^{2}=0.65$, *df* = 1, p=0.4201, Figure 3B).

Direct pairwise comparison of Kaplan–Meier survival curves between infected groups revealed that although cumulative mortality was higher in insects challenged with the High dose than in those receiving the Low dose, their overall survival trajectories did not differ significantly over the 16-day monitoring window ($\chi^{2}=0.96$, *df* = 1, *p* = 0.327; Figure 3C, Table S6).

## 4. Discussion

The present methodological study provides better tools for the standardization of procedures for the experimental TrV infections of triatomine bugs, as well as more precise measurement for monitoring viral loads in infected insects. Our experiments using *R. prolixus* as a model exemplify how these tools could be used for the analysis of the susceptibility and monitoring of other triatomines of epidemiological importance in Chagas disease transmission.

Virus quantification using TrV-specific primers in a qPCR-based system allowed a departure from the purely qualitative approaches that long dominated triatomine virology [8,22,39]. This tool allowed the measurement of what was previously a technical blind spot: the precise viral load and replication dynamics required to uniformly standardize inocula [8,21,22,23]. This assay could also facilitate investigations on feral triatomine TrV infections and could automate surveillance of infected bugs using this virus in control interventions.

A critical requirement for surveillance assays is the avoidance of false positives arising from co-occurring organisms, as cross-reactivity has been documented in *T. cruzi* molecular diagnostics and specificity must therefore be demonstrated against non-target triatomines and related parasites [40,41,42]. Although our assay showed analytical specificity with no cross-reactivity in the tested controls, broader prospective field validation remains necessary because wild triatomines harbour more diverse microbiota than laboratory colonies, which can affect real-world assay performance [43,44].

As a starting point, the optimized TrV purification protocol (EGM) increased viral particle yield compared to the previous workflows of Marti et al. (2008) [8] and Susevich et al. (2017) [9]. Securing a good level of viral biomass is essential for designing an efficient, standardized evaluation of the susceptibility of TrV in other regional triatomines that actively maintain *T. cruzi* transmission [2,3,4].

Although TrV exhibits strict host taxonomic specificity toward the subfamily Triatominae [15] and has been detected across 19 different *T. cruzi* vectors (*Meccus longipennis*, *Mepraia gajardoi*, *Mepraia parapatrica*, *Mepraia spinolai*, *Psammolestes coreodes*, *Rhodnius neglectus*, *R. prolixus*, *Triatoma brasiliensis*, *Triatoma breyeri*, *Triatoma delpontei*, *Triatoma dimidiata*, *Triatoma guasayana*, *T. infestans*, *Triatoma maculata*, *Triatoma pallidipennis*, *Triatoma patagonica*, *Triatoma platensis*, *Triatoma rubrovaria*, *Triatoma sordida*) [5,8,9,17,18,22,23,44,45,46,47,48,49,50,51,52,53,54,55,56], but the susceptibility of other putative targets for control is required before the use of this control strategy could be considered. Within this context, our study using *R. prolixus* presents a guiding example; while previously detected as a baseline infection within laboratory insectaries [20,23], functional data regarding its active replication were missing. Because homologous cell lines for in vitro titration are unavailable [20,57], evaluating TrV infection across triatomine species relies entirely on robust in vivo viral load quantification. This limitation highlights the value of the comprehensive protocol presented here to track viral loads and replication kinetics under laboratory conditions. In this regard, our comprehensive protocol successfully captured the characteristic biphasic trajectory of intestinal viral kinetics. Crucially, the subsequent surge in viral titers observed across both dosage groups confirms productive intracellular replication, demonstrating that the infection progresses beyond the initial ingested inoculum. Nevertheless, interpreting these early kinetic profiles requires acknowledging an inherent limitation of our detection strategy. Specifically, targeting total genomic RNA (+ssRNA) by qPCR cannot differentiate the residual viral inoculum from newly synthesized viral strands during early infection stages (1–3 dpi), making it difficult to pinpoint the exact onset of active viral replication. However, as residual inoculum decays or clears over time, subsequent significant increases in viral load above the initial levels confirm productive viral cycles. Dissecting these early entry dynamics from active replication machinery via strand-specific qPCR targeting negative-sense RNA (−ssRNA) intermediates represents a key parameter for future mechanistic investigations. However, for prospective applications of TrV as a biocontrol agent against other triatomine species, establishing the precise timing of viral replication initiation is not strictly required, as confirming overall productive replication and mortality remains sufficient for vector control evaluations.

On the other hand, in this study, only the high dose significantly compromised *R. prolixus* survival, whereas the low dose had no observable effect on its viability. Although cohort size limitations from sequential sampling restricted viral load tracking beyond 12 dpi in the low-dose group, daily monitoring through 16 dpi confirmed zero mortality after 14 dpi, indicating a stable, non-lethal equilibrium. Furthermore, direct comparison between infected cohorts revealed no significant difference in survival trajectories, indicating that a higher viral dose does not accelerate host death but merely increases mortality sufficiently to separate from controls. Indicating that the virus’s efficacy cannot be uniformly assumed across other species; each potential host may possess distinct susceptibility thresholds, and specific replication kinetics must be characterized individually to prevent management failures in such biological control programs.

Calibrating the dosage is non-negotiable before moving toward field trials or large-scale applications. The applied inoculum must consistently clear the threshold required to trigger high mortality and delayed moulting [11,19,58]. Securing this initial biological effect is what unlocks horizontal transmission via coprophagy, allowing the virus to disperse autonomously throughout breeding and aggregation sites, but quantifying TrV dissemination within triatomine conglomerates (either by coprophagy or cannibalism) awaits further studies. These quantitative datasets would provide the foundations to turn TrV into a reliable, measurable biocontrol tool—a pressing necessity as vector populations build up genetic resistance to standard chemical insecticides [5,6]. However, translated biological effects must be interpreted considering that physical genome counts determined by qPCR represent total viral particles, including an undetermined proportion of non-infectious virions or defective genomes. While TrV presents a highly desirable biosecurity profile due to its strict host specificity restricted exclusively to triatomines without risk to vertebrates or non-target fauna [15], the broader application of Dicistroviruses as biopesticides faces inherent limitations. These include potential sublethal persistence, variability in horizontal transmission dynamics, and the long-term risk of host resistance development, all of which require careful evaluation during dosage calibration. Rigorous infectivity validation and workflow standardization are the baseline requirements to ensure safety and efficacy in the integrated management of these neglected vectors [1,4].

## 5. Conclusions

Our results demonstrate that *Triatoma virus* infection kinetics and replication dynamics in *R. prolixus* monitoring are influenced by a combination of methodological and physiological factors (e.g., purification efficiency and initial inoculum size), and that a high viral dose successfully clears the threshold required to compromise host survival. We documented the precise in vivo replication profiles of TrV and suggested that its quantification and dose-calibration may influence the tracking of vector susceptibility and horizontal transmission. These insights lay the groundwork for future studies exploring functional interactions between host and pathogens across other epidemiologically significant triatomines, and open the door to standardized, virus-based strategies for biological control.

## Data Availability

The data presented in this study are available within the article and its Supplementary Files (Supplementary_data.zip). The complete R v4.2.1 pipeline utilized for statistical analysis and data visualization is available as Supplementary data (TrV_viral_infection_Rp_R_Analysis/trv_purification_infection_kinetics.R). The Sanger consensus nucleotide sequences validated in this study are provided as FASTA files within the Supplementary Data (Fastas_Sequencing_TrV folder).

## Supplementary Materials

The following supporting information can be downloaded at https://www.mdpi.com/article/10.3390/v18090962/s1: Figure S1: Duplicate verification of TrV end-point PCR products; Table S1: Operational parameters for the four TrV isolation and purification methods; Table S2: Comparative analysis of *Triatoma virus* (TrV) purification efficiency and absolute quantification across different methods; Table S3: Experimental groups, inoculum compositions, and estimated viral doses per insect; Table S4: Statistical evaluation of viral replication or purification efficiency across different experimental groups; Table S5: Pairwise statistical comparison of viral load kinetics between high-dose and low-dose experimental cohorts; Table S6: Summary statistics of Log-Rank (Mantel-Cox) test for survival comparisons.

## Abbreviations

The following abbreviations are used in this manuscript:

dpi	Days post-inoculation
EGM	Everardo Gutiérrez-Millán (optimized purification method)
PCR	Polymerase chain reaction
qPCR	Quantitative polymerase chain reaction
TrV	*Triatoma virus*
*TrVgp1*	Nonstructural protein precursor
*TrVgp2*	Capsid protein precursor
