# Supplementary material for: Improved Purification and Quantitative Kinetics Monitoring of Triatoma virus Infection of Rhodnius prolixus
Source: Viruses. 2026 Sep 2;18(9):962. doi: 10.3390/v18090962 (PMC13611934; doi:10.3390/v18090962)
Supplement: Supplementary file 1 [file viruses-18-00962-s001.zip › Supplementary data/qPCR_Reports/Reports_S1/Report_S1.0_Quant_and_Melt_reports_TrVgp1_standar_curve.pdf]

QIAGEN

www.qiagen.com

Quantitation Report

Experiment Information

Run Name	qPCR_TrVgp1_standar_curve
Run Start	26/11/2025 01:44:34 p. m.
Run Finish	26/11/2025 03:43:51 p. m.
Operator	Ever
Notes	
Run On Software Version	Rotor-Gene Q Software 2.3.5.1
Run Signature	The Run Signature is valid.
Gain Green	9.67
Machine Serial No.	0317130

Quantitation Information

Threshold	0.0697
Left Threshold	1.000
Standard Curve Imported	No
Standard Curve (1)	conc= 10^(-0.302*CT + 11.708)
Standard Curve (2)	CT = -3.314*log(conc) + 38.803
Reaction efficiency (*)	(* = 10^(-1/m) - 1) 1.00319
M	-3.3143
B	38.80329
R Value	0.9931
R^2 Value	0.98624
Start normalising from cycle	1
Noise Slope Correction	No
No Template Control Threshold	% 0
Reaction Efficiency Threshold	Disabled
Normalisation Method	Dynamic Tube Normalisation
Digital Filter	Light

(Continued on next page)...

Sample Page	Page 1
Imported Analysis Settings	

Quantitation data for Cycling A.Green

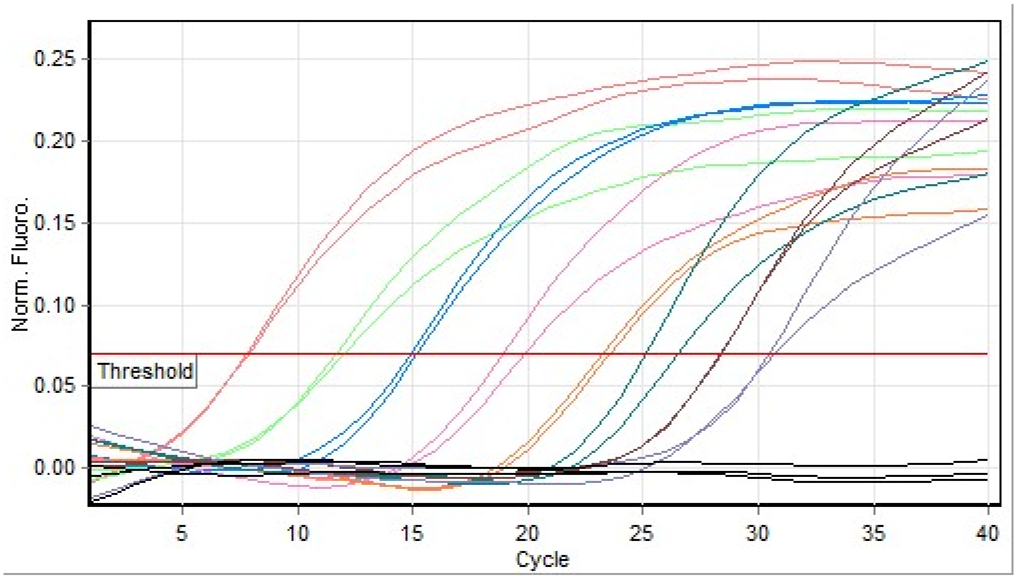

Standard Curve

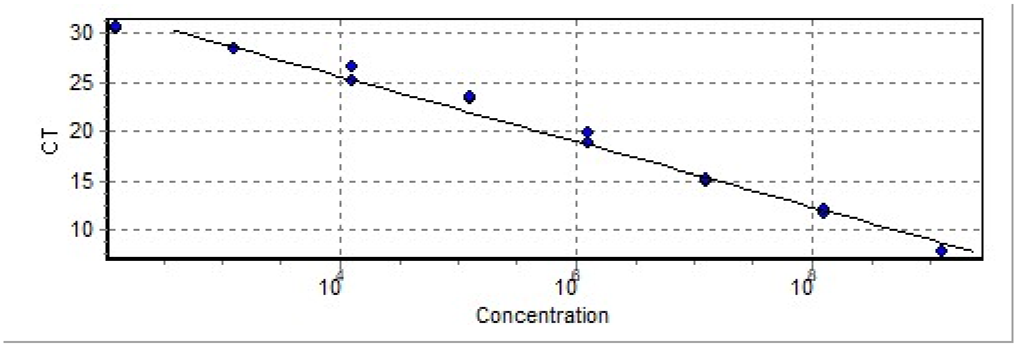

No.	Color	Name	Type	Ct	Ct Comment	Given Conc (Copies)	Calc Conc (Copies)
A1		D1_1	Standard	7.75		1.24E+09	2.34E+09
A3		D1_3	Standard	7.82		1.24E+09	2.23E+09
A4		D2_1	Standard	11.64		1.24E+08	1.57E+08

(Continued on next page)...

No.	Color	Name	Type	Ct	Ct Comment	Given Conc (Copies)	Calc Conc (Copies)
A6		D2_3	Standard	11.99		1.24E+08	1.23E+08
A7		D3_1	Standard	14.90		1.24E+07	1.63E+07
A8		D3_2	Standard	15.18		1.24E+07	1.34E+07
B2		D4_1	Standard	18.90		1.24E+06	1.01E+06
B4		D4_3	Standard	19.85		1.24E+06	5.22E+05
B5		D5_1	Standard	23.22		1.24E+05	5.02E+04
B7		D5_3	Standard	23.54		1.24E+05	4.03E+04
B8		D6_1	Standard	26.50		1.24E+04	5.17E+03
C1		D6_2	Standard	25.12		1.24E+04	1.34E+04
C3		D7_1	Standard	28.33		1.24E+03	1.44E+03
C5		D7_3	Standard	28.36		1.24E+03	1.41E+03
C6		D8_1	Standard	30.45		1.24E+02	3.31E+02
C8		D8_3	Standard	30.68		1.24E+02	2.83E+02
I6		negative 1	NTC				
I7		negative 2	NTC				
I8		negative 3	NTC				

Legend:
NEG (NTC) - Sample cancelled due to NTC Threshold.
NEG (R. Eff) - Sample cancelled as efficiency less than reaction efficiency threshold.

This report was generated by Rotor-Gene Q Series Software 2.3.5 (Build 1)
Copyright © 2013 QIAGEN GmbH. All Rights Reserved.

QIAGEN

www.qiagen.com

Melt Report

Experiment Information

Run Name	qPCR_TrVgp1_standar_curve
Run Start	26/11/2025 01:44:34 p. m.
Run Finish	26/11/2025 03:43:51 p. m.
Operator	Ever
Notes	
Run On Software Version	Rotor-Gene Q Software 2.3.5.1
Run Signature	The Run Signature is valid.
Gain Green	9.67
Machine Serial No.	0317130

Melt Information

Digital Filter	Light
Imported Analysis Settings	
Sample Page	Page 1
Temp. Threshold	0°C
Threshold	0.

Profile

Cycle	Cycle Point
Hold 1	Hold @ 95°C, 10min 0s
Cycling (40 repeats)	Step 1: Hold @ 95°C, 30s
	Step 2: Hold @ 58°C, 30s
	Step 3: Hold @ 72°C, 30s, acquiring to Cycling A([Green][1][1])
Hold 2	Hold @ 72°C, 10min 0s
Melt	Ramp from 60°C to 95°C
	Hold for 90s on the 1st step
	Hold for 5s on next steps,Melt A([Green][1][1])

Melt data for Melt A.Green

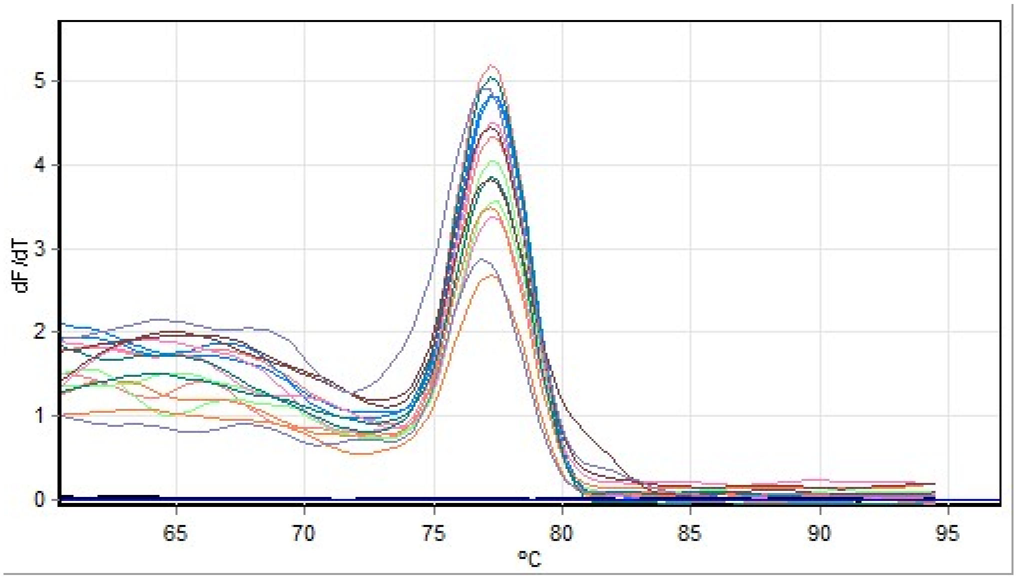

No.	Color	Name	Genotype	Peak 1	Peak 2	Peak 3	Peak 4
A1		D1_1		61.8	66.5	77.2	83.5
A3		D1_3		66.0	77.3	85.0	91.5
A4		D2_1		65.0	77.3	85.0	90.5
A6		D2_3		61.5	67.0	77.3	85.7
A7		D3_1		61.2	66.0	77.3	85.5
A8		D3_2		67.0	77.3	82.3	86.7

(Continued on next page)...

No.	Color	Name	Genotype	Peak 1	Peak 2	Peak 3	Peak 4
B2		D4_1		65.2	69.5	77.3	82.5
B4		D4_3		63.8	77.3	84.5	89.8
B5		D5_1		62.5	66.5	72.5	77.2
B7		D5_3		63.5	77.2	82.2	86.5
B8		D6_1		64.2	77.3	82.5	85.7
C1		D6_2		64.3	77.2		
C3		D7_1		66.0	77.2	88.2	
C5		D7_3		64.8	77.2	88.2	
C6		D8_1		64.5	68.0	77.0	88.8
C8		D8_3		63.3	67.8	72.3	77.0
I6		negative 1		63.0	69.5	74.5	80.5
I7		negative 2		68.5	73.8	77.5	84.2
I8		negative 3		63.7	68.7	73.0	77.3

Bin Name	Temperature	Sample No.	Sample Name	Peak

This report was generated by Rotor-Gene Q Series Software 2.3.5 (Build 1)
Copyright © 2013 QIAGEN GmbH. All Rights Reserved.
