# Supplementary material for: Improved Purification and Quantitative Kinetics Monitoring of Triatoma virus Infection of Rhodnius prolixus
Source: Viruses. 2026 Sep 2;18(9):962. doi: 10.3390/v18090962 (PMC13611934; doi:10.3390/v18090962)
Supplement: Supplementary file 1 [file viruses-18-00962-s001.zip › Supplementary data/qPCR_Reports/Reports_S2/Report_S2.0_Quant_and_Melt_reports_TrVgp1_High_and_Low_doses.pdf]

QIAGEN

www.qiagen.com

Quantitation Report

Experiment Information

Run Name	qPCR_TrVgp1_doses
Run Start	03/12/2024 04:49:39 p. m.
Run Finish	03/12/2024 06:39:44 p. m.
Operator	Ever
Notes	
Run On Software Version	Rotor-Gene Q Software 2.3.1.49
Run Signature	The Run Signature is valid.
Gain Green	8.67
Machine Serial No.	0317130

Quantitation Information

Threshold	0.0697
Left Threshold	1.000
Standard Curve Imported	Yes
Standard Curve (1)	conc= 10^(-0.302*CT + 11.708)
Standard Curve (2)	CT = -3.314*log(conc) + 38.803
Reaction efficiency (*)	(* = 10^(-1/m) - 1) 1.00319
Start normalising from cycle	1
Noise Slope Correction	No
No Template Control Threshold	% 0
Reaction Efficiency Threshold	Disabled
Normalisation Method	Dynamic Tube Normalisation
Digital Filter	Light
Sample Page	Page 1
Imported Analysis Settings	

Quantitation data for Cycling A.Green

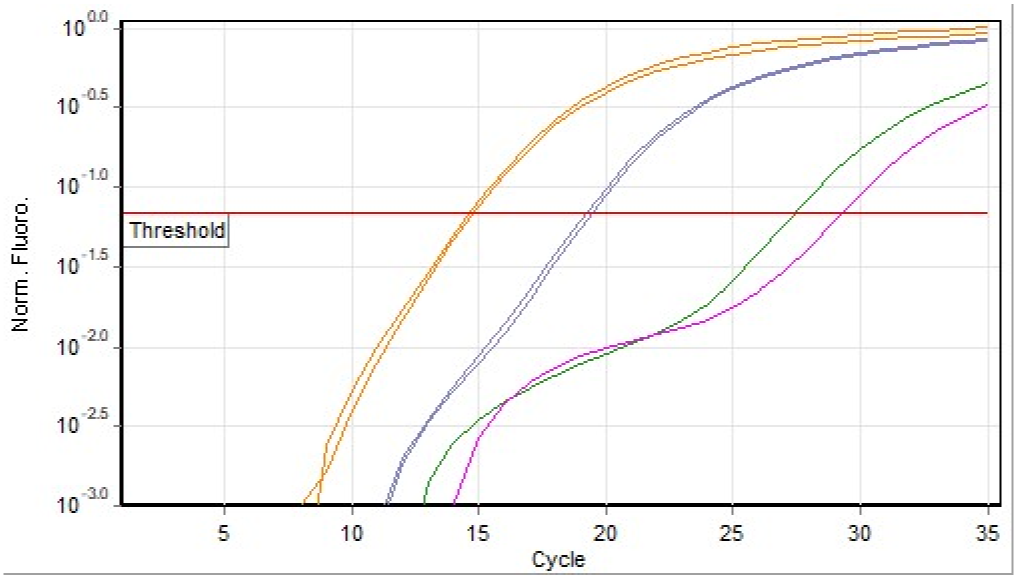

Standard Curve

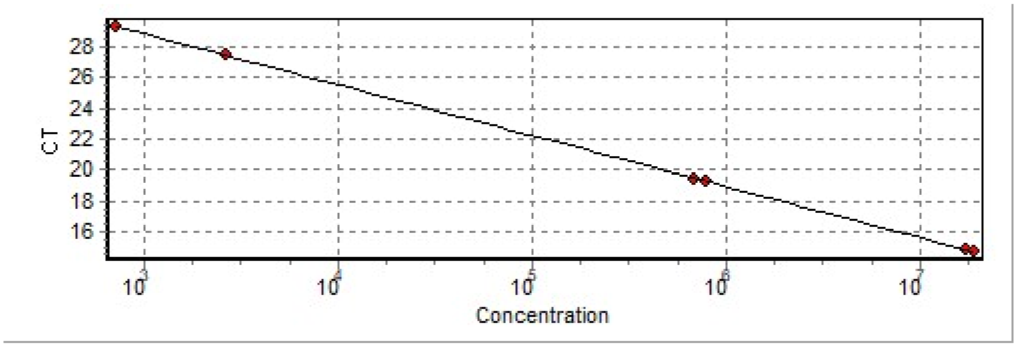

No.	Color	Name	Type	Ct	Ct Comment	Given Conc (Copies)	Calc Conc (Copies)
A1		High dose rep 1	Unknown	14.83			17144127.8965
A2		High dose rep 2	Unknown	14.69			18835218.8887
A3		Low dose rep 1	Unknown	19.25			793575.145295
A4		Low dose rep 2	Unknown	19.46			687895.516206
F1		neg_TrVgp1	NTC	27.44			2683.52060019
F2		neg_TrVgp1	NTC	29.33			720.300588897

Legend:
NEG (NTC) - Sample cancelled due to NTC Threshold.
NEG (R. Eff) - Sample cancelled as efficiency less than reaction efficiency threshold.

This report was generated by Rotor-Gene Q Series Software 2.3.5 (Build 1)
Copyright © 2013 QIAGEN GmbH. All Rights Reserved.

www.qiagen.com

Melt Report

Experiment Information

Run Name	qPCR_TrVgp1_doses
Run Start	03/12/2024 04:49:39 p. m.
Run Finish	03/12/2024 06:39:44 p. m.
Operator	Ever
Notes	
Run On Software Version	Rotor-Gene Q Software 2.3.1.49
Run Signature	The Run Signature is valid.
Gain Green	8.67
Machine Serial No.	0317130

Melt Information

Digital Filter	Light
Imported Analysis Settings	
Sample Page	Page 1
Temp. Threshold	0°C
Threshold	0.

Profile

Cycle	Cycle Point
Hold 1	Hold @ 95°C, 10min 0s
Cycling (35 repeats)	Step 1: Hold @ 95°C, 30s
	Step 2: Hold @ 58°C, 30s
	Step 3: Hold @ 72°C, 30s, acquiring to Cycling A([Green][1][1])
Hold 2	Hold @ 72°C, 10min 0s
Melt	Ramp from 60°C to 95°C
	Hold for 90s on the 1st step
	Hold for 5s on next steps,Melt A([Green][1][1])

Melt data for Melt A.Green

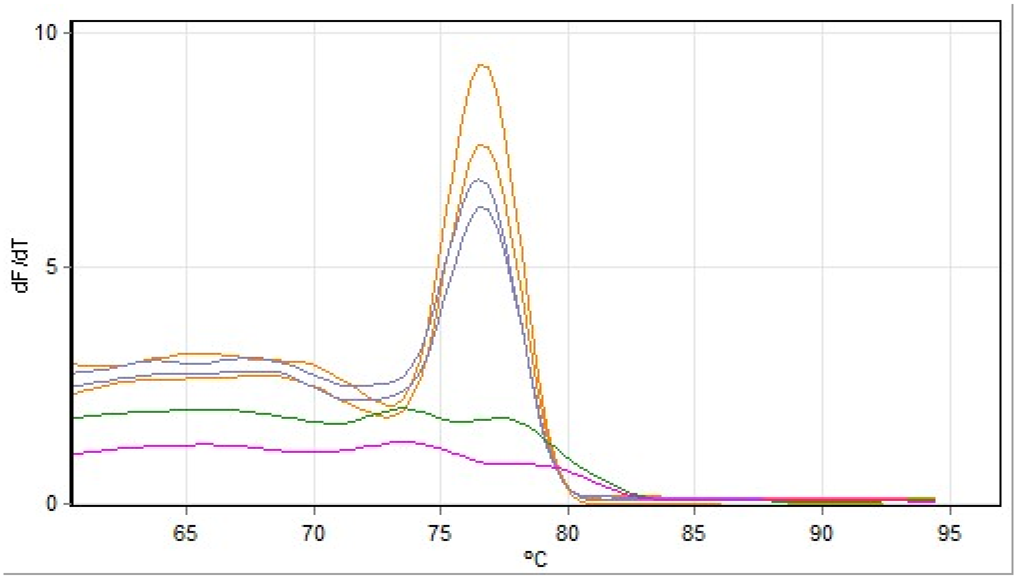

No.	Color	Name	Genotype	Peak 1	Peak 2	Peak 3	Peak 4
A1		High dose rep 1		68.0	76.5	81.8	87.0
A2		High dose rep 2		65.5	76.5		
A3		Low dose rep 1		63.7	67.5	76.5	86.5
A4		Low dose rep 2		64.8	67.7	76.5	81.7
F1		neg_TrVgp1		65.8	73.5	77.2	86.2
F2		neg_TrVgp1		65.7	73.5	78.0	89.5

Bin Name	Temperature	Sample No.	Sample Name	Peak

This report was generated by Rotor-Gene Q Series Software 2.3.5 (Build 1)
Copyright © 2013 QIAGEN GmbH. All Rights Reserved.
