# Supplementary material for: Improved Purification and Quantitative Kinetics Monitoring of Triatoma virus Infection of Rhodnius prolixus
Source: Viruses. 2026 Sep 2;18(9):962. doi: 10.3390/v18090962 (PMC13611934; doi:10.3390/v18090962)
Supplement: Supplementary file 1 [file viruses-18-00962-s001.zip › Supplementary data/qPCR_Reports/Reports_S3/Report_S3.0_Quant_and_Melt_Tcruzi_Strains_and_Infected_Tpallidipennis_Samples.pdf]

www.qiagen.com

Quantitation Report

Experiment Information

Run Name	Tcruzi_Strains_and_Infected_Tpallidipennis_Samples
Run Start	07/08/2026 10:29:54 a. m.
Run Finish	07/08/2026 12:12:43 p. m.
Operator	Ever
Notes	
Run On Software Version	Rotor-Gene Q Software 2.3.5.1
Run Signature	The Run Signature is valid.
Gain Green	9.67
Machine Serial No.	0317130

Quantitation data for Cycling A.Green

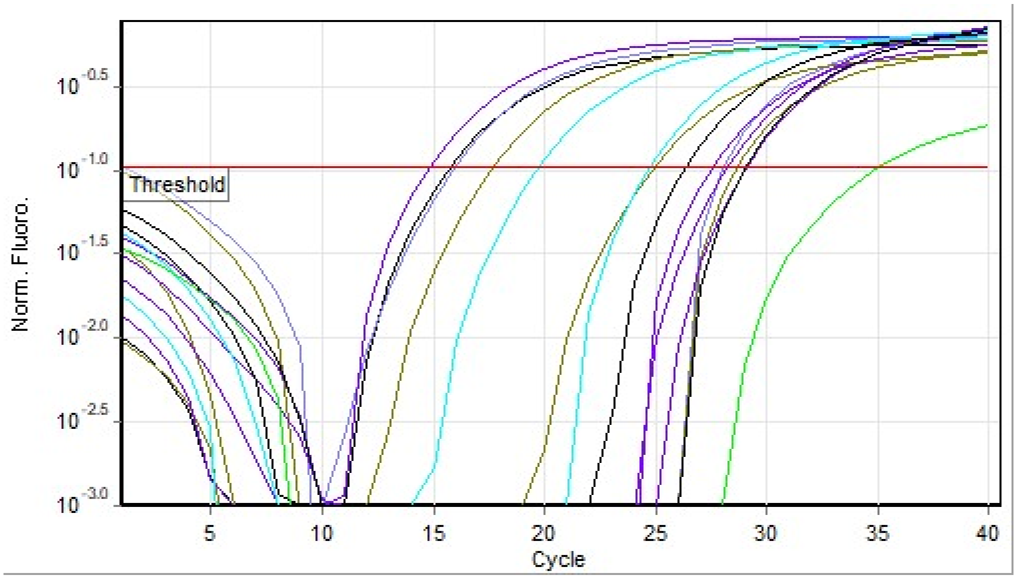

No.	Color	Name	Type	Ct	Ct Comment	Given Conc (Copies)	Calc Conc (Copies)
A1	■	Trypanosoma cruzi strain Tc19	Unknown	15.73			

(Continued on next page)...

No.	Color	Name	Type	Ct	Ct Comment	Given Conc (Copies)	Calc Conc (Copies)
A2		Trypanosoma cruzi strain Chalcatzingo	Unknown	15.96			
A3		Trypanosoma cruzi strain Chilpancingo	Unknown	17.67			
A4		Trypanosoma cruzi strain Morelos	Unknown	14.83			
A5		Trypanosoma cruzi strain Valentina	Unknown	19.75			
B4		Trypanosoma cruzi strain Chalcatzingo Tpallidipennis	Unknown	28.05			
B5		Trypanosoma cruzi strain Chilpancingo Tpallidipennis	Unknown	28.65			
B6		Trypanosoma cruzi strain Chilpancingo Tpallidipennis	Unknown	24.91			
B7		Trypanosoma cruzi strain Morelos Tpallidipennis	Unknown	27.55			
B8		Trypanosoma cruzi strain Morelos Tpallidipennis	Unknown	29.09			
C1		Trypanosoma cruzi strain Morelos Tpallidipennis	Unknown	28.26			
C3		Trypanosoma cruzi strain Valentina Tpallidipennis	Unknown	24.71			
C4		Trypanosoma cruzi strain Tc19 Tpallidipennis	Unknown	29.05			
C5		Trypanosoma cruzi strain Tc19 Tpallidipennis	Unknown	26.34			
F4		negative Tcruzi	NTC	35.03			

Legend:
NEG (NTC) - Sample cancelled due to NTC Threshold.
NEG (R. Eff) - Sample cancelled as efficiency less than reaction efficiency threshold.

This report was generated by Rotor-Gene Q Series Software 2.3.5 (Build 1)
Copyright © 2013 QIAGEN GmbH. All Rights Reserved.

QIAGEN

www.qiagen.com

Melt Report

Experiment Information

Run Name	Tcruzi_Strains_and_Infected_Tpallidipennis_Samples
Run Start	07/08/2026 10:29:54 a. m.
Run Finish	07/08/2026 12:12:43 p. m.
Operator	Ever
Notes	
Run On Software Version	Rotor-Gene Q Software 2.3.5.1
Run Signature	The Run Signature is valid.
Gain Green	9.67
Machine Serial No.	0317130

Melt Information

Digital Filter	Light
Imported Analysis Settings	
Sample Page	Page 1
Temp. Threshold	0°C
Threshold	0.

Profile

Cycle	Cycle Point
Hold	Hold @ 95°C, 10min 0s
Cycling (40 repeats)	Step 1: Hold @ 95°C, 15s
	Step 2: Hold @ 60°C, 60s, acquiring to Cycling A([Green][1][1])
Melt	Ramp from 60°C to 95°C
	Hold for 90s on the 1st step
	Hold for 5s on next steps,Melt A([Green][1][1])

Melt data for Melt A.Green

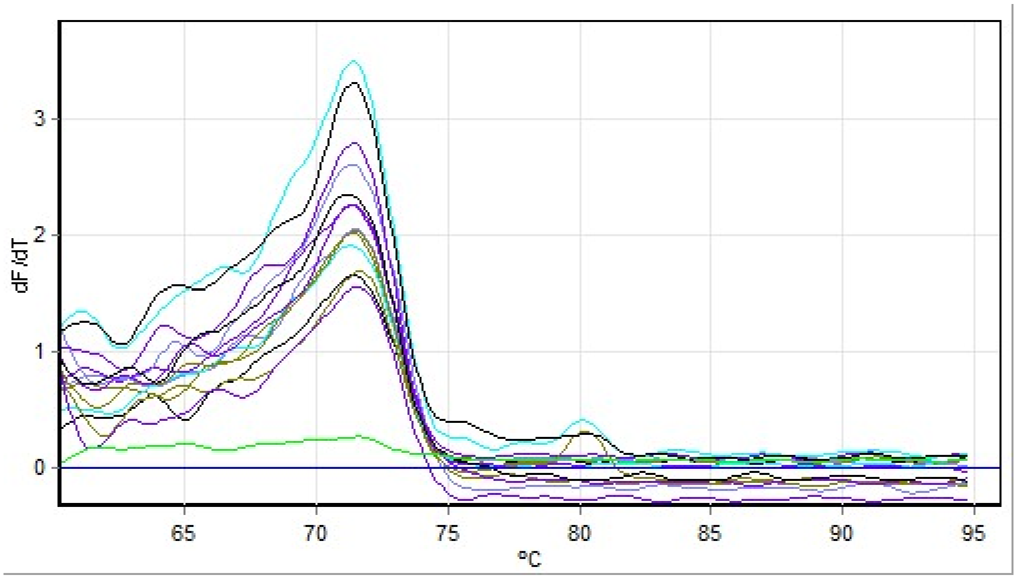

No.	Color	Name	Genotype	Peak 1	Peak 2	Peak 3	Peak 4
A1		Trypanosoma cruzi strain Tc19		61.25	63.75	71.40	76.65
A2		Trypanosoma cruzi strain Chalcatzingo		61.50	65.40	67.40	71.50
A3		Trypanosoma cruzi strain Chilpancingo		61.10	62.60	71.35	78.00
A4		Trypanosoma cruzi strain Morelos		61.25	63.75	71.40	78.25
A5		Trypanosoma cruzi strain Valentina		60.65	67.10	71.25	78.90
B4		Trypanosoma cruzi strain Chalcatzingo Tpallidipennis		64.65	71.35		
B5		Trypanosoma cruzi strain Chilpancingo Tpallidipennis		64.90	67.10	71.65	
B6		Trypanosoma cruzi strain Chilpancingo Tpallidipennis		63.25	65.25	71.50	77.90
B7		Trypanosoma cruzi strain Morelos Tpallidipennis		63.00	66.25	71.50	
B8		Trypanosoma cruzi strain Morelos Tpallidipennis		62.65	65.25	71.35	
C1		Trypanosoma cruzi strain Morelos Tpallidipennis		64.25	71.40	76.10	79.15
C3		Trypanosoma cruzi strain Valentina Tpallidipennis		61.00	66.50	71.40	75.25
C4		Trypanosoma cruzi strain Tc19 Tpallidipennis		61.15	64.75	71.40	75.25
C5		Trypanosoma cruzi strain Tc19 Tpallidipennis		62.90	71.15		
F4		negative Tcruzi		61.75	65.00	71.60	77.15

Bin Name	Temperature	Sample No.	Sample Name	Peak

This report was generated by Rotor-Gene Q Series Software 2.3.5 (Build 1)
Copyright © 2013 QIAGEN GmbH. All Rights Reserved.
