# Supplementary material for: Improved Purification and Quantitative Kinetics Monitoring of Triatoma virus Infection of Rhodnius prolixus
Source: Viruses. 2026 Sep 2;18(9):962. doi: 10.3390/v18090962 (PMC13611934; doi:10.3390/v18090962)
Supplement: Supplementary file 1 [file viruses-18-00962-s001.zip › Supplementary data/qPCR_Reports/Reports_S3/Report_S3.1_Quant_Melt_Tcruzi_Strains_and_Infected_Tpallidipennis_Samples_vs_TrV.pdf]

QIAGEN

www.qiagen.com

Quantitation Report

Experiment Information

Run Name	Tcruzi_strains_and_samples_Tcruzi_infected_vs_TrV
Run Start	06/08/2026 10:14:00 a. m.
Run Finish	06/08/2026 12:12:45 p. m.
Operator	Ever
Notes	
Run On Software Version	Rotor-Gene Q Software 2.3.5.1
Run Signature	The Run Signature is valid.
Gain Green	9.
Machine Serial No.	0317130

Quantitation Information

Threshold	0.0697
Left Threshold	1.000
Standard Curve Imported	No
Standard Curve (1)	conc= 10^(-0.302*CT + 11.708)
Standard Curve (2)	CT = -3.314*log(conc) + 38.803
Reaction efficiency (*)	(* = 10^(-1/m) - 1) 1.00319
M	-3.3143
B	38.80329
R Value	0.9931
R^2 Value	0.98624
Start normalising from cycle	1
Noise Slope Correction	No
No Template Control Threshold	% 0
Reaction Efficiency Threshold	Disabled
Normalisation Method	Dynamic Tube Normalisation
Digital Filter	Light

Quantitation data for Cycling A.Green

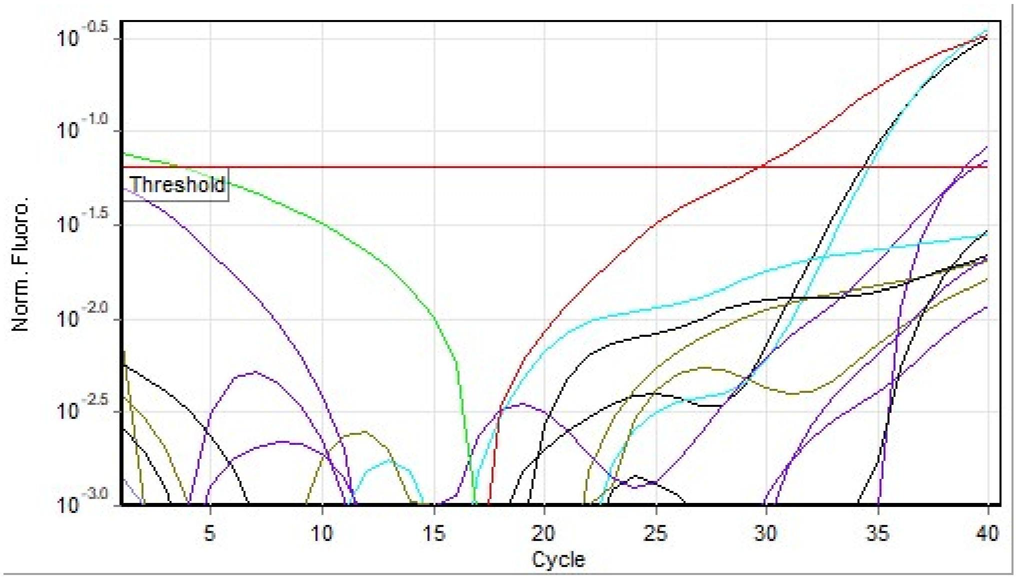

Standard Curve

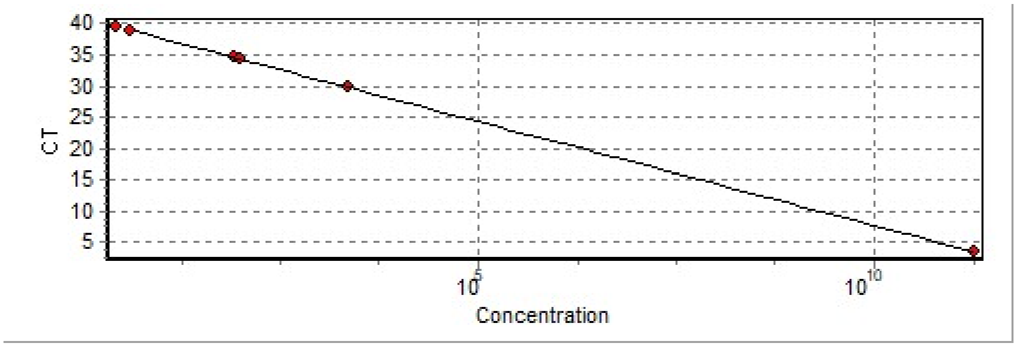

No.	Color	Name	Type	Ct	Ct Comment	Given Conc (Copies)	Calc Conc (Copies)
A1		Trypanosoma cruzi strain Tc19	Unknown	34.29			97.3982243621
A2		Trypanosoma cruzi strain Chalcatzingo	Unknown				
A3		Trypanosoma cruzi strain Chilpancingo	Unknown				
A4		Trypanosoma cruzi strain Morelos	Unknown	38.92			3.91378932478
A5		Trypanosoma cruzi strain Valentina	Unknown	34.57			79.8405917617
B4		Trypanosoma cruzi strain Chalcatzingo Tpallidipennis	Unknown				

(Continued on next page)...

No.	Color	Name	Type	Ct	Ct Comment	Given Conc (Copies)	Calc Conc (Copies)
B5		Trypanosoma cruzi strain Chilpancingo Tpallidipennis	Unknown				
B6		Trypanosoma cruzi strain Chilpancingo Tpallidipennis	Unknown				
B7		Trypanosoma cruzi strain Morelos Tpallidipennis	Unknown				
B8		Trypanosoma cruzi strain Morelos Tpallidipennis	Unknown				
C1		Trypanosoma cruzi strain Morelos Tpallidipennis	Unknown	39.48			2.64955782981
C3		Trypanosoma cruzi strain Valentina Tpallidipennis	Unknown				
C4		Trypanosoma cruzi strain Tc19 Tpallidipennis	Unknown				
C5		Trypanosoma cruzi strain Tc19 Tpallidipennis	Unknown				
C7		positive TrV	Positive Control	29.76			2253.80896185
E1		negative TrV	NTC	3.53			183872721723.

Legend:
NEG (NTC) - Sample cancelled due to NTC Threshold.
NEG (R. Eff) - Sample cancelled as efficiency less than reaction efficiency threshold.

This report was generated by Rotor-Gene Q Series Software 2.3.5 (Build 1)
Copyright © 2013 QIAGEN GmbH. All Rights Reserved.

www.qiagen.com

Melt Report

Experiment Information

Run Name	Tcruzi_strains_and_samples_Tcruzi_infected_vs_TrV
Run Start	06/08/2026 10:14:00 a. m.
Run Finish	06/08/2026 12:12:45 p. m.
Operator	Ever
Notes	
Run On Software Version	Rotor-Gene Q Software 2.3.5.1
Run Signature	The Run Signature is valid.
Gain Green	9.
Machine Serial No.	0317130

Melt Information

Digital Filter	Light
Imported Analysis Settings	
Sample Page	Page 1
Temp. Threshold	0°C
Threshold	0.

Profile

Cycle	Cycle Point
Hold 1	Hold @ 95°C, 10min 0s
Cycling (40 repeats)	Step 1: Hold @ 95°C, 30s
	Step 2: Hold @ 58°C, 30s
	Step 3: Hold @ 72°C, 30s, acquiring to Cycling A([Green][1][1])
Hold 2	Hold @ 72°C, 10min 0s
Melt	Ramp from 60°C to 95°C
	Hold for 90s on the 1st step
	Hold for 5s on next steps,Melt A([Green][1][1])

Melt data for Melt A.Green

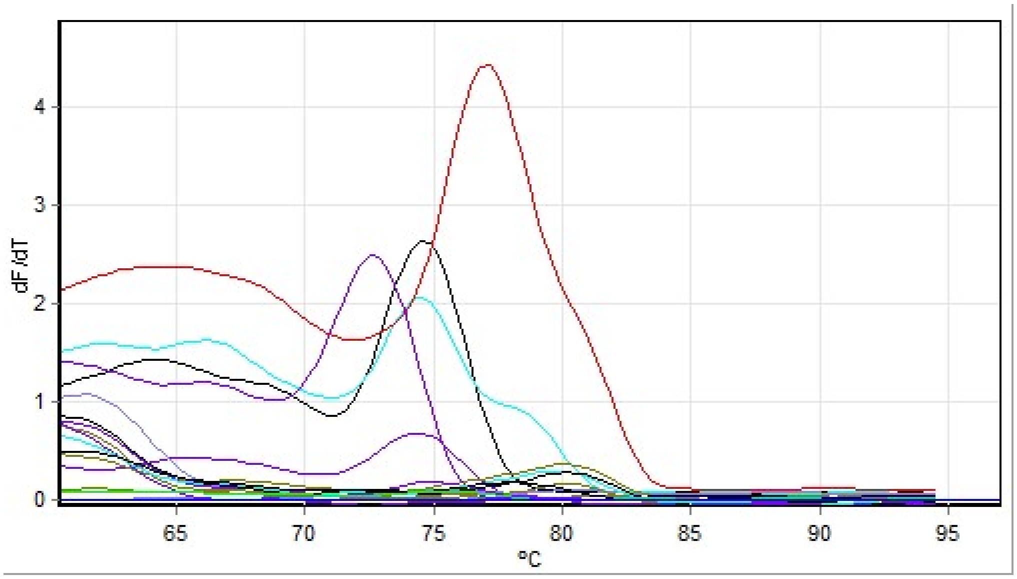

No.	Color	Name	Genotype	Peak 1	Peak 2	Peak 3	Peak 4
A1		Trypanosoma cruzi strain Tc19		64.0	74.5	84.8	89.7
A2		Trypanosoma cruzi strain Chalcatzingo		64.7	69.7	73.5	77.0
A3		Trypanosoma cruzi strain Chilpancingo		61.8	66.2	70.7	76.5
A4		Trypanosoma cruzi strain Morelos		65.5	74.3	79.5	
A5		Trypanosoma cruzi strain Valentina		62.2	66.0	74.5	82.7
B4		Trypanosoma cruzi strain Chalcatzingo Tpallidipennis		61.5	75.7	80.5	84.5

(Continued on next page)...

No.	Color	Name	Genotype	Peak 1	Peak 2	Peak 3	Peak 4
B5		Trypanosoma cruzi strain Chilpancingo Tpallidipennis		76.5	80.2	86.7	
B6		Trypanosoma cruzi strain Chilpancingo Tpallidipennis		67.2	80.0	86.5	91.2
B7		Trypanosoma cruzi strain Morelos Tpallidipennis		68.7	78.7		
B8		Trypanosoma cruzi strain Morelos Tpallidipennis		74.8	82.7	88.7	
C1		Trypanosoma cruzi strain Morelos Tpallidipennis		66.0	72.7	82.2	84.2
C3		Trypanosoma cruzi strain Valentina Tpallidipennis		72.3	79.7		
C4		Trypanosoma cruzi strain Tc19 Tpallidipennis		72.5	80.2	86.7	
C5		Trypanosoma cruzi strain Tc19 Tpallidipennis		61.0	71.0	78.0	85.0
C7		positive TrV		63.8	65.0	77.0	90.5
E1		negative TrV		64.5	69.2	75.5	83.3

Bin Name	Temperature	Sample No.	Sample Name	Peak

This report was generated by Rotor-Gene Q Series Software 2.3.5 (Build 1)
Copyright © 2013 QIAGEN GmbH. All Rights Reserved.
