# Supplementary material for: Improved Purification and Quantitative Kinetics Monitoring of Triatoma virus Infection of Rhodnius prolixus
Source: Viruses. 2026 Sep 2;18(9):962. doi: 10.3390/v18090962 (PMC13611934; doi:10.3390/v18090962)
Supplement: Supplementary file 1 [file viruses-18-00962-s001.zip › Supplementary data/qPCR_Reports/Reports_S3/Report_S3.2_Quant_and_Melt_report_Wild_and_laboratory_bugs_vs_TrV.pdf]

QIAGEN

www.qiagen.com

Quantitation Report

Experiment Information

Run Name	qPCR_Wild_and_Laboratory_bugs_vs_TrV
Run Start	24/04/2025 10:55:25 a. m.
Run Finish	24/04/2025 12:58:43 p. m.
Operator	
Notes	
Run On Software Version	Rotor-Gene Q Software 2.3.1.49
Run Signature	The Run Signature is valid.
Gain Green	9.67
Machine Serial No.	0317130

Quantitation Information

Threshold	0.0697
Left Threshold	1.000
Standard Curve Imported	No
Standard Curve (1)	conc= 10^(-0.302*CT + 11.708)
Standard Curve (2)	CT = -3.314*log(conc) + 38.803
Reaction efficiency (*)	(* = 10^(-1/m) - 1) 1.00319
M	-3.3143
B	38.80329
R Value	0.9931
R^2 Value	0.98624
Start normalising from cycle	1
Noise Slope Correction	No
No Template Control Threshold	% 0
Reaction Efficiency Threshold	Disabled
Normalisation Method	Dynamic Tube Normalisation
Digital Filter	Light

Quantitation data for Cycling A.Green

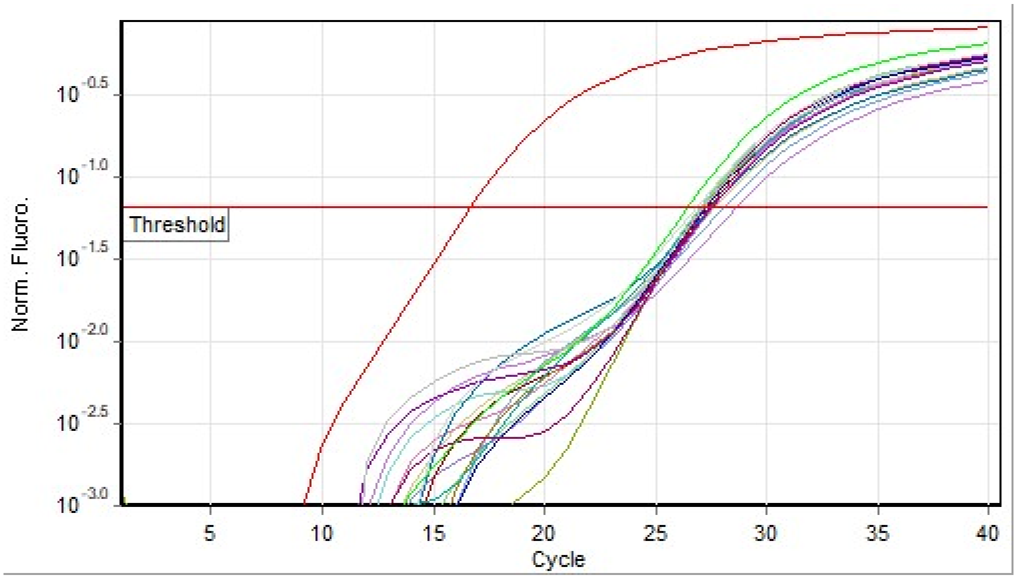

Standard Curve

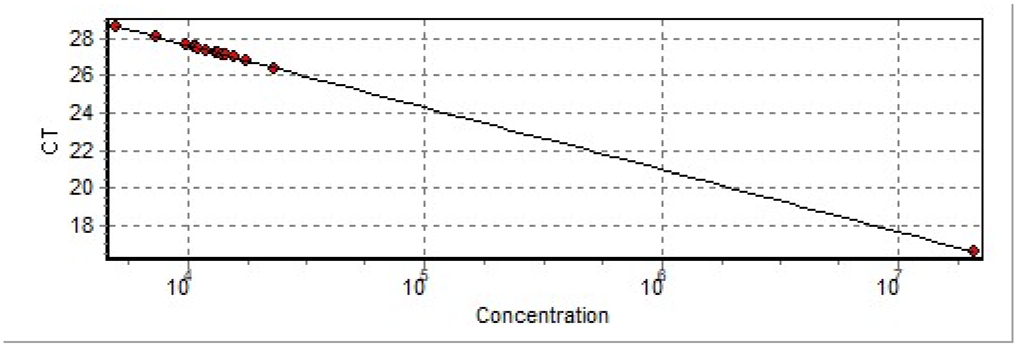

No.	Color	Name	Type	Ct	Ct Comment	Given Conc (ng/ul)	Calc Conc (ng/ul)
E8		positive TrV	Positive Control	16.62			20825158.0995
F5		Wild Tpallidipennis	Unknown	27.64			9877.08077859
F6		Wild Tpallidipennis	Unknown	26.97			15703.5965620
F7		Wild Tpallidipennis	Unknown	27.07			14595.9417535
F8		Wild Tpallidipennis	Unknown	28.06			7362.44647900
G1		Wild Tpallidipennis	Unknown	27.54			10598.1247761
G2		Wild Tpallidipennis	Unknown	28.62			4987.32099509

(Continued on next page)...

No.	Color	Name	Type	Ct	Ct Comment	Given Conc (ng/ul)	Calc Conc (ng/ul)
G3		Rhodnius prolixus	Unknown	27.52			10725.9012027
G4		Rhodnius prolixus	Unknown	27.36			11932.6868703
G5		Rhodnius prolixus	Unknown	27.22			13149.6677589
G6		Rhodnius prolixus	Unknown	27.14			13987.6976324
G7		Rhodnius prolixus	Unknown	27.15			13878.2982992
G8		Rhodnius prolixus	Unknown	27.47			11118.5482294
H1		Triatoma phyllosoma	Unknown	27.23			13071.1078498
H2		Triatoma phyllosoma	Unknown	27.50			10861.9492228
H3		Triatoma phyllosoma	Unknown	27.19			13424.2030038
H4		Triatoma phyllosoma	Unknown	26.79			17728.6199604
H5		Triatoma phyllosoma	Unknown	27.13			14036.9182301
I1		negative TrV	NTC	26.41			23158.2251671

Legend:
NEG (NTC) - Sample cancelled due to NTC Threshold.
NEG (R. Eff) - Sample cancelled as efficiency less than reaction efficiency threshold.

This report was generated by Rotor-Gene Q Series Software 2.3.5 (Build 1)
Copyright © 2013 QIAGEN GmbH. All Rights Reserved.

QIAGEN

www.qiagen.com

Melt Report

Experiment Information

Run Name	qPCR_TrV_INF_0_1_5_7_9_12_muertas_24_04_2025
Run Start	24/04/2025 10:55:25 a. m.
Run Finish	24/04/2025 12:58:43 p. m.
Operator	
Notes	
Run On Software Version	Rotor-Gene Q Software 2.3.1.49
Run Signature	The Run Signature is valid.
Gain Green	9.67
Machine Serial No.	0317130

Melt Information

Digital Filter	Light
Imported Analysis Settings	
Sample Page	Page 1
Temp. Threshold	0°C
Threshold	0.

Profile

Cycle	Cycle Point
Hold 1	Hold @ 95°C, 10min 0s
Cycling (40 repeats)	Step 1: Hold @ 95°C, 30s
	Step 2: Hold @ 58°C, 30s
	Step 3: Hold @ 72°C, 30s, acquiring to Cycling A([Green][1][1])
Hold 2	Hold @ 72°C, 10min 0s
Melt	Ramp from 60°C to 95°C
	Hold for 90s on the 1st step
	Hold for 5s on next steps,Melt A([Green][1][1])

Melt data for Melt A.Green

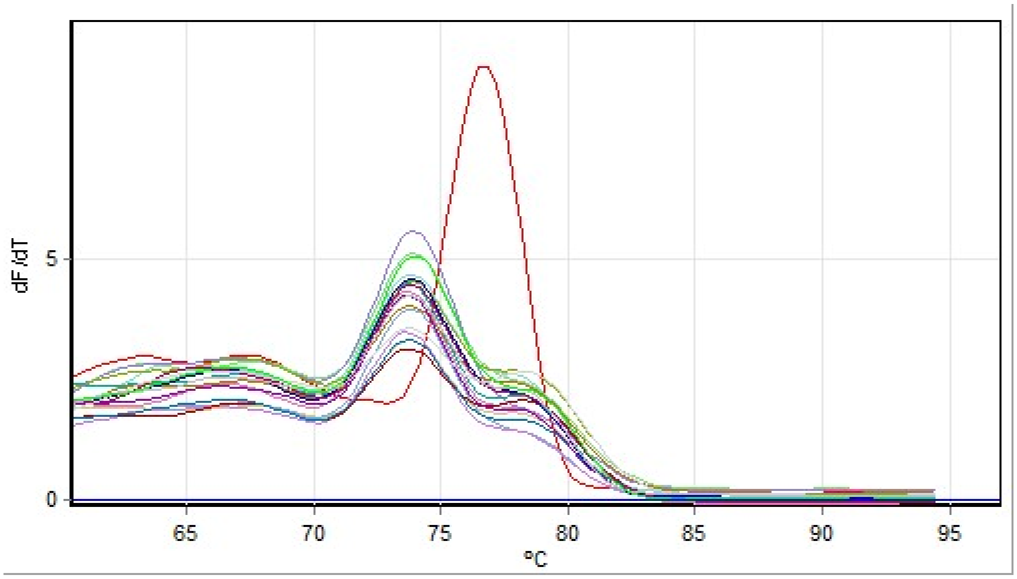

No.	Color	Name	Genotype	Peak 1	Peak 2	Peak 3	Peak 4
E8		positive TrV		63.3	67.3	76.7	81.5
F5		Wild Tpallidipennis		61.5	67.5	73.8	77.8
F6		Wild Tpallidipennis		63.5	67.7	74.0	90.3
F7		Wild Tpallidipennis		64.7	73.8	90.5	
F8		Wild Tpallidipennis		67.2	73.8	90.3	
G1		Wild Tpallidipennis		63.8	66.8	74.0	87.3

(Continued on next page)...

No.	Color	Name	Genotype	Peak 1	Peak 2	Peak 3	Peak 4
G2		Wild Tpallidipennis		65.5	73.7	87.3	91.5
G3		Rhodnius prolixus		66.5	73.7		
G4		Rhodnius prolixus		62.3	67.0	73.7	78.2
G5		Rhodnius prolixus		63.7	67.2	73.8	93.0
G6		Rhodnius prolixus		63.8	67.2	74.0	78.0
G7		Rhodnius prolixus		67.0	73.7	78.0	88.8
G8		Rhodnius prolixus		66.8	73.7	88.8	
H1		Triatoma phyllosoma		66.5	73.8	91.8	
H2		Triatoma phyllosoma		66.3	73.7	77.7	88.5
H3		Triatoma phyllosoma		65.5	73.8		
H4		Triatoma phyllosoma		66.3	73.7	78.3	85.5
H5		Triatoma phyllosoma		66.5	73.8	92.3	93.8
I1		negative TrV		66.7	74.0	86.0	90.2

Bin Name	Temperature	Sample No.	Sample Name	Peak

This report was generated by Rotor-Gene Q Series Software 2.3.5 (Build 1)
Copyright © 2013 QIAGEN GmbH. All Rights Reserved.
