# Supplementary material for: Improved Purification and Quantitative Kinetics Monitoring of Triatoma virus Infection of Rhodnius prolixus
Source: Viruses. 2026 Sep 2;18(9):962. doi: 10.3390/v18090962 (PMC13611934; doi:10.3390/v18090962)
Supplement: Supplementary file 1 [file viruses-18-00962-s001.zip › Supplementary data/qPCR_Reports/Reports_S4/Report_S4.0_Quant_and_Melt_report_TrVgp1_HighDose_1dpi.pdf]

www.qiagen.com

Quantitation Report

Experiment Information

Run Name	qPCR_TrV_HD_1dpi
Run Start	23/03/2026 11:12:52 a. m.
Run Finish	23/03/2026 01:11:42 p. m.
Operator	Ever
Notes	
Run On Software Version	Rotor-Gene Q Software 2.3.5.1
Run Signature	The Run Signature is valid.
Gain Green	9.33
Machine Serial No.	0317130

Quantitation Information

Threshold	0.0697
Left Threshold	1.000
Standard Curve Imported	No
Standard Curve (1)	conc= 10^(-0.302*CT + 11.708)
Standard Curve (2)	CT = -3.314*log(conc) + 38.803
Reaction efficiency (*)	(* = 10^(-1/m) - 1) 1.00319
M	-3.3143
B	38.80329
R Value	0.9931
R^2 Value	0.98624
Start normalising from cycle	1
Noise Slope Correction	No
No Template Control Threshold	% 0
Reaction Efficiency Threshold	Disabled
Normalisation Method	Dynamic Tube Normalisation
Digital Filter	Light

Quantitation data for Cycling A.Green

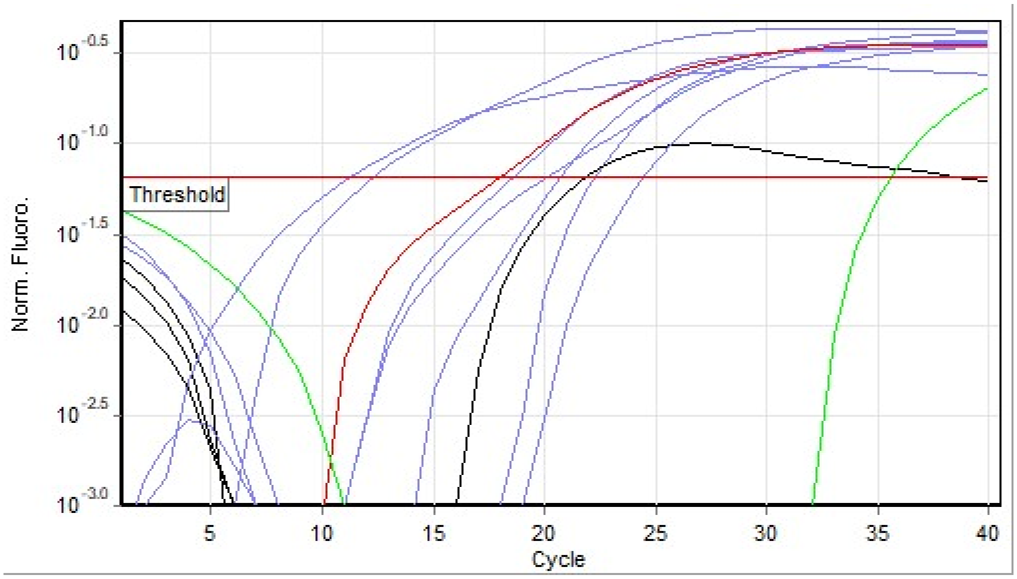

Standard Curve

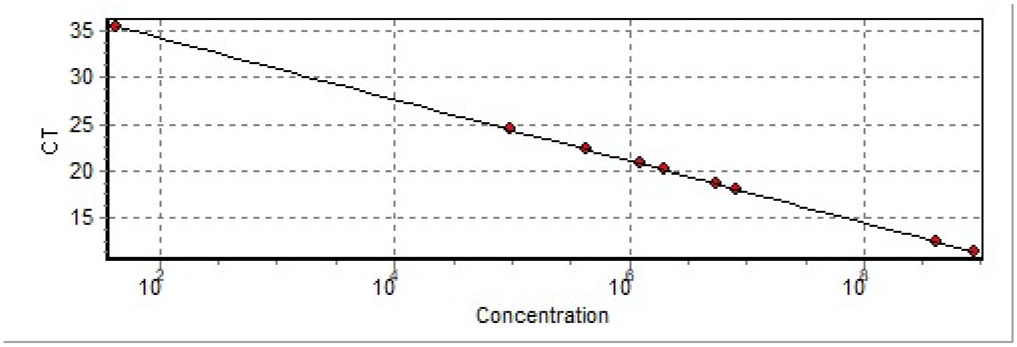

No.	Color	Name	Type	Ct	Ct Comment	Given Conc (copies/ul)	Calc Conc (copies/ul)
A1		TrV_HD_1dpi	Unknown				
A2		TrV_HD_1dpi	Unknown	22.24			420945.098671
A3		TrV_HD_1dpi	Unknown		NEG (Multi Ct)		
A4		TrV_HD_1dpi	Unknown	12.29			419181791.758
A5		TrV_HD_1dpi	Unknown	18.53			5540111.94587
A6		TrV_HD_1dpi	Unknown	20.68			1239577.44273

(Continued on next page)...

No.	Color	Name	Type	Ct	Ct Comment	Given Conc (copies/ul)	Calc Conc (copies/ul)
A7		TrV_HD_1dpi	Unknown				
A8		TrV_HD_1dpi	Unknown	24.38			95005.3444333
B1		TrV_HD_1dpi	Unknown	11.22			881348688.488
B2		TrV_HD_1dpi	Unknown	20.03			1948183.47927
D6		positive TrV	Positive Control	17.95			8253574.76804
H1		negative TrV	NTC	35.53			41.5282724036

Legend:
NEG (NTC) - Sample cancelled due to NTC Threshold.
NEG (R. Eff) - Sample cancelled as efficiency less than reaction efficiency threshold.

This report was generated by Rotor-Gene Q Series Software 2.3.5 (Build 1)
Copyright © 2013 QIAGEN GmbH. All Rights Reserved.

QIAGEN

www.qiagen.com

Melt Report

Experiment Information

Run Name	qPCR_TrV_HD_1dpi
Run Start	23/03/2026 11:12:52 a. m.
Run Finish	23/03/2026 01:11:42 p. m.
Operator	Ever
Notes	
Run On Software Version	Rotor-Gene Q Software 2.3.5.1
Run Signature	The Run Signature is valid.
Gain Green	9.33
Machine Serial No.	0317130

Melt Information

Digital Filter	Light
Imported Analysis Settings	
Sample Page	Page 1
Temp. Threshold	0°C
Threshold	0.

Profile

Cycle	Cycle Point
Hold 1	Hold @ 95°C, 10min 0s
Cycling (40 repeats)	Step 1: Hold @ 95°C, 30s
	Step 2: Hold @ 58°C, 30s
	Step 3: Hold @ 72°C, 30s, acquiring to Cycling A([Green][1][1])
Hold 2	Hold @ 72°C, 10min 0s
Melt	Ramp from 60°C to 95°C
	Hold for 90s on the 1st step
	Hold for 5s on next steps,Melt A([Green][1][1])

Melt data for Melt A.Green

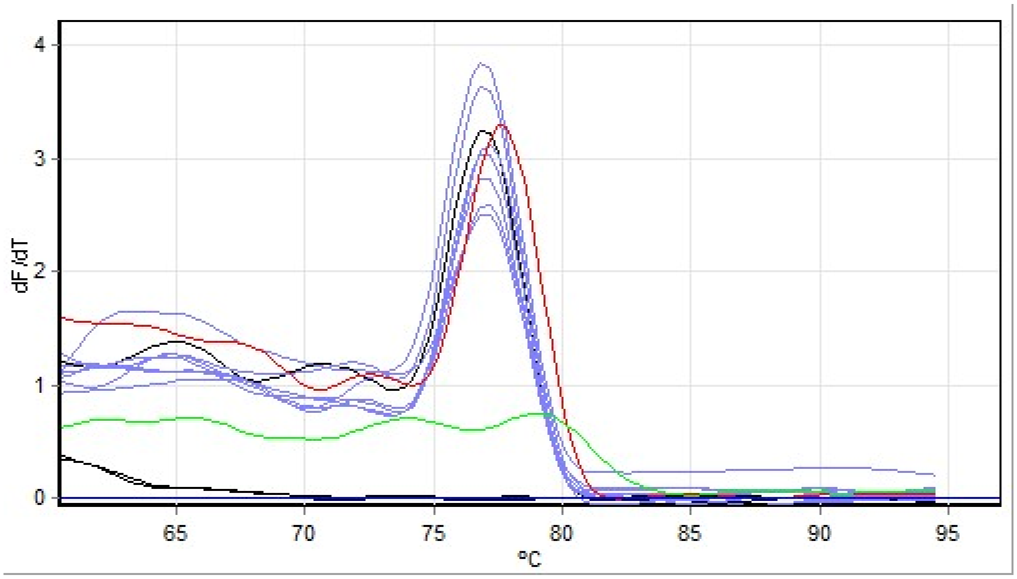

No.	Color	Name	Genotype	Peak 1	Peak 2	Peak 3	Peak 4
A1		TrV_HD_1dpi		65.5	73.5	80.3	
A2		TrV_HD_1dpi		63.5	77.0	81.7	90.5
A3		TrV_HD_1dpi		65.0	70.8	77.0	85.3
A4		TrV_HD_1dpi		65.0	71.8	77.0	
A5		TrV_HD_1dpi		61.5	65.5	71.5	77.0
A6		TrV_HD_1dpi		64.3	77.2	84.7	90.2

(Continued on next page)...

No.	Color	Name	Genotype	Peak 1	Peak 2	Peak 3	Peak 4
A7		TrV_HD_1dpi		73.5	78.0	84.2	89.3
A8		TrV_HD_1dpi		62.3	64.8	77.0	84.3
B1		TrV_HD_1dpi		66.2	77.0	83.7	88.0
B2		TrV_HD_1dpi		64.7	72.2	77.0	81.8
D6		positive TrV		63.2	72.5	77.5	85.2
H1		negative TrV		62.5	65.5	74.0	79.0

Bin Name	Temperature	Sample No.	Sample Name	Peak

This report was generated by Rotor-Gene Q Series Software 2.3.5 (Build 1)
Copyright © 2013 QIAGEN GmbH. All Rights Reserved.
