# Supplementary material for: Improved Purification and Quantitative Kinetics Monitoring of Triatoma virus Infection of Rhodnius prolixus
Source: Viruses. 2026 Sep 2;18(9):962. doi: 10.3390/v18090962 (PMC13611934; doi:10.3390/v18090962)
Supplement: Supplementary file 1 [file viruses-18-00962-s001.zip › Supplementary data/qPCR_Reports/Reports_S4/Report_S4.1_Quant_and_Melt_report_TrVgp1_HighDose_3_5_7_9dpi.pdf]

QIAGEN

www.qiagen.com

Quantitation Report

Experiment Information

Run Name	qPCR_TrV_HD_3_5_7_9dpi
Run Start	02/04/2026 09:20:20 a. m.
Run Finish	02/04/2026 11:20:47 a. m.
Operator	Ever
Notes	
Run On Software Version	Rotor-Gene Q Software 2.3.5.1
Run Signature	The Run Signature is valid.
Gain Green	8.67
Machine Serial No.	0317130

Quantitation Information

Threshold	0.0697
Left Threshold	1.000
Standard Curve Imported	No
Standard Curve (1)	conc= 10^(-0.302*CT + 11.708)
Standard Curve (2)	CT = -3.314*log(conc) + 38.803
Reaction efficiency (*)	(* = 10^(-1/m) - 1) 1.00319
M	-3.3143
B	38.80329
R Value	0.9931
R^2 Value	0.98624
Start normalising from cycle	1
Noise Slope Correction	No
No Template Control Threshold	% 0
Reaction Efficiency Threshold	Disabled
Normalisation Method	Dynamic Tube Normalisation
Digital Filter	Light

Quantitation data for Cycling A.Green

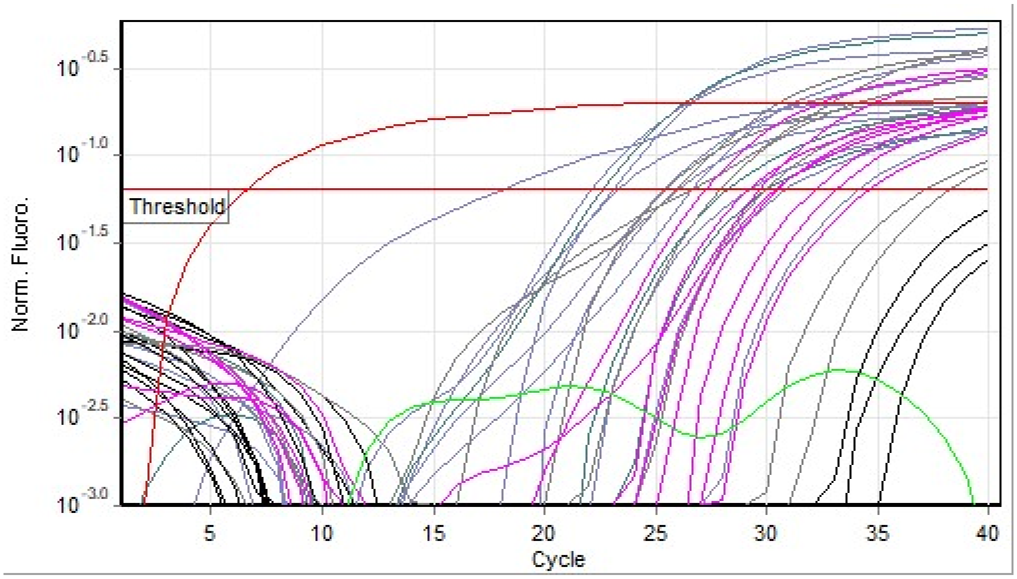

Standard Curve

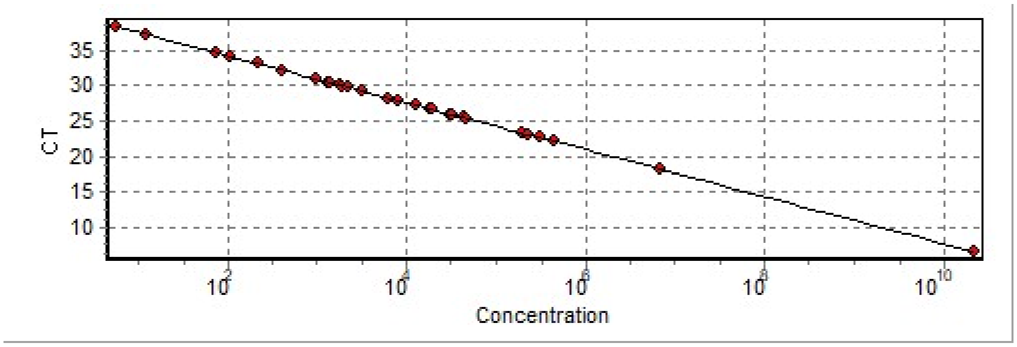

No.	Color	Name	Type	Ct	Ct Comment	Given Conc (copies/ul)	Calc Conc (copies/ul)
A1		TrV_HD_3dpi	Unknown	30.96			980.1121
A2		TrV_HD_3dpi	Unknown	18.20			6,914,328.6860
A3		TrV_HD_3dpi	Unknown	29.27			3,173.3800
A4		TrV_HD_3dpi	Unknown	26.01			30,615.4310
A5		TrV_HD_3dpi	Unknown	23.08			233,377.0447
A6		TrV_HD_3dpi	Unknown	34.18			105.1800
A7		TrV_HD_3dpi	Unknown	25.38			47,424.6354

(Continued on next page)...

No.	Color	Name	Type	Ct	Ct Comment	Given Conc (copies/ul)	Calc Conc (copies/ul)
A8		TrV_HD_3dpi	Unknown	26.67			19,367.8984
B1		TrV_HD_3dpi	Unknown	23.30			201,033.2057
B2		TrV_HD_3dpi	Unknown	22.16			444,028.0255
B3		TrV_HD_5dpi	Unknown	30.10			1,781.5419
B4		TrV_HD_5dpi	Unknown	28.29			6,293.7796
B5		TrV_HD_5dpi	Unknown				
B6		TrV_HD_5dpi	Unknown				
B7		TrV_HD_5dpi	Unknown				
B8		TrV_HD_5dpi	Unknown	22.64			318,173.5701
C1		TrV_HD_5dpi	Unknown				
C2		TrV_HD_5dpi	Unknown				
C3		TrV_HD_5dpi	Unknown				
C4		TrV_HD_5dpi	Unknown				
C5		TrV_HD_7dpi	Unknown	37.26			12.3361
C6		TrV_HD_7dpi	Unknown	26.72			18,714.8394
C7		TrV_HD_7dpi	Unknown				
C8		TrV_HD_7dpi	Unknown	27.94			7,977.0117
D1		TrV_HD_7dpi	Unknown				
D2		TrV_HD_7dpi	Unknown	29.99			1,926.2682
D3		TrV_HD_7dpi	Unknown	38.37			5.7313
D4		TrV_HD_7dpi	Unknown	25.94			32,052.5112
D5		TrV_HD_7dpi	Unknown				
D6		TrV_HD_7dpi	Unknown	25.47			44,592.5474
D7		TrV_HD_9dpi	Unknown				
D8		TrV_HD_9dpi	Unknown	29.74			2,284.7557
E1		TrV_HD_9dpi	Unknown	30.47			1,382.2883
E2		TrV_HD_9dpi	Unknown	29.27			3,170.7583
E3		TrV_HD_9dpi	Unknown	29.11			12,840.8860
E4		TrV_HD_9dpi	Unknown	32.19			418.1947
E5		TrV_HD_9dpi	Unknown	30.53			1,325.7807
E6		TrV_HD_9dpi	Unknown	33.12			218.9381

(Continued on next page)...

No.	Color	Name	Type	Ct	Ct Comment	Given Conc (copies/ul)	Calc Conc (copies/ul)
E7		TrV_HD_9dpi	Unknown	34.67			74.5471
E8		TrV_HD_9dpi	Unknown				
F1		positive TrV	Unknown	6.58			22,189,405,850
F2		negative TrV	Unknown				

Legend:
NEG (NTC) - Sample cancelled due to NTC Threshold.
NEG (R. Eff) - Sample cancelled as efficiency less than reaction efficiency threshold.

This report was generated by Rotor-Gene Q Series Software 2.3.5 (Build 1)
Copyright © 2013 QIAGEN GmbH. All Rights Reserved.

QIAGEN

www.qiagen.com

Melt Report

Experiment Information

Run Name	qPCR_TrV_HD_3_5_7_9dpi
Run Start	02/04/2026 09:20:20 a. m.
Run Finish	02/04/2026 11:20:47 a. m.
Operator	Ever
Notes	
Run On Software Version	Rotor-Gene Q Software 2.3.5.1
Run Signature	The Run Signature is valid.
Gain Green	8.67
Machine Serial No.	0317130

Melt Information

Digital Filter	Light
Imported Analysis Settings	
Sample Page	Page 1
Temp. Threshold	0°C
Threshold	0.

Profile

Cycle	Cycle Point
Hold 1	Hold @ 95°C, 10min 0s
Cycling (40 repeats)	Step 1: Hold @ 95°C, 30s
	Step 2: Hold @ 58°C, 30s
	Step 3: Hold @ 72°C, 30s, acquiring to Cycling A([Green][1][1])
Hold 2	Hold @ 72°C, 10min 0s
Melt	Ramp from 60°C to 95°C
	Hold for 90s on the 1st step
	Hold for 5s on next steps,Melt A([Green][1][1])

Melt data for Melt A.Green

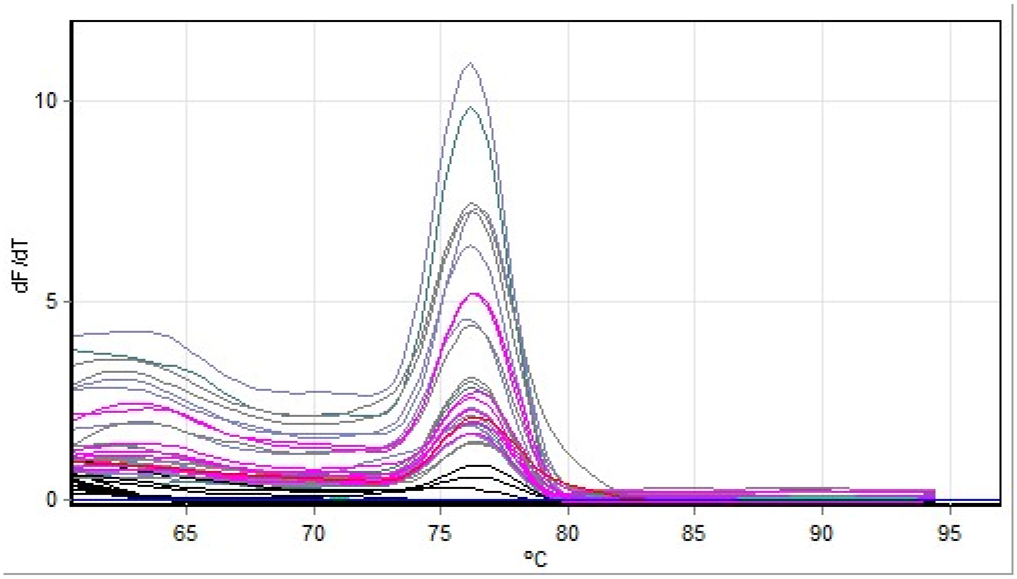

No.	Color	Name	Genotype	Peak 1	Peak 2	Peak 3	Peak 4
A1		TrV_HD_3dpi		61.0	67.0	76.2	83.5
A2		TrV_HD_3dpi		60.8	67.5	76.0	83.3
A3		TrV_HD_3dpi		62.7	70.7	76.2	81.0
A4		TrV_HD_3dpi		63.0	70.8	76.2	81.5
A5		TrV_HD_3dpi		63.0	70.3	76.2	81.0
A6		TrV_HD_3dpi		61.8	70.5	76.0	82.8

(Continued on next page)...

No.	Color	Name	Genotype	Peak 1	Peak 2	Peak 3	Peak 4
A7		TrV_HD_3dpi		63.0	76.0	80.8	85.3
A8		TrV_HD_3dpi		61.8	76.2	84.8	90.3
B1		TrV_HD_3dpi		61.8	71.8	76.2	84.5
B2		TrV_HD_3dpi		62.2	71.0	76.5	81.7
B3		TrV_HD_5dpi		65.5	71.5	76.2	
B4		TrV_HD_5dpi		62.2	76.3	81.0	85.5
B5		TrV_HD_5dpi					
B6		TrV_HD_5dpi		64.5			
B7		TrV_HD_5dpi					
B8		TrV_HD_5dpi		71.0	76.2		
C1		TrV_HD_5dpi		75.3			
C2		TrV_HD_5dpi					
C3		TrV_HD_5dpi		77.8	86.7	91.0	
C4		TrV_HD_5dpi		76.5			
C5		TrV_HD_7dpi		65.0	71.7	76.5	89.3
C6		TrV_HD_7dpi		62.2	76.2	83.8	88.7
C7		TrV_HD_7dpi					
C8		TrV_HD_7dpi		63.2	76.3	84.0	88.5
D1		TrV_HD_7dpi		67.3	76.3	85.3	91.0
D2		TrV_HD_7dpi		61.7	76.2	82.7	91.0
D3		TrV_HD_7dpi		61.2	76.5	87.2	92.2
D4		TrV_HD_7dpi		62.3	76.2	85.5	91.5
D5		TrV_HD_7dpi					
D6		TrV_HD_7dpi		62.3	76.2	80.7	85.3
D7		TrV_HD_9dpi					
D8		TrV_HD_9dpi		63.0	76.3	84.5	89.2
E1		TrV_HD_9dpi		63.0	68.2	76.3	83.7
E2		TrV_HD_9dpi		76.2			
E3		TrV_HD_9dpi		63.5	76.3	82.5	89.0
E4		TrV_HD_9dpi		63.0	76.5	82.8	86.7
E5		TrV_HD_9dpi		62.8	70.3	76.2	80.7

(Continued on next page)...

No.	Color	Name	Genotype	Peak 1	Peak 2	Peak 3	Peak 4
E6		TrV_HD_9dpi		62.3	76.2	80.5	84.8
E7		TrV_HD_9dpi		63.2	72.3	76.2	80.7
E8		TrV_HD_9dpi		68.5	72.7	75.5	79.8
F1		positive TrV		76.5	89.5		
F2		negative TrV		71.0	81.3	88.2	91.3

Bin Name	Temperature	Sample No.	Sample Name	Peak

This report was generated by Rotor-Gene Q Series Software 2.3.5 (Build 1)
Copyright © 2013 QIAGEN GmbH. All Rights Reserved.
