# Supplementary material for: Improved Purification and Quantitative Kinetics Monitoring of Triatoma virus Infection of Rhodnius prolixus
Source: Viruses. 2026 Sep 2;18(9):962. doi: 10.3390/v18090962 (PMC13611934; doi:10.3390/v18090962)
Supplement: Supplementary file 1 [file viruses-18-00962-s001.zip › Supplementary data/qPCR_Reports/Reports_S4/Report_S4.2_Quant_and_Melt_report_TrVgp1_HighDose_12_14_16dpi.pdf]

www.qiagen.com

Quantitation Report

Experiment Information

Run Name	qPCR_TrV_HD_12_14_16dpi
Run Start	13/04/2026 09:42:04 a. m.
Run Finish	13/04/2026 11:41:23 a. m.
Operator	Ever
Notes	
Run On Software Version	Rotor-Gene Q Software 2.3.5.1
Run Signature	The Run Signature is valid.
Gain Green	9.33
Machine Serial No.	0317130

Quantitation Information

Threshold	0.0697
Left Threshold	1.000
Standard Curve Imported	No
Standard Curve (1)	conc= 10^(-0.302*CT + 11.708)
Standard Curve (2)	CT = -3.314*log(conc) + 38.803
Reaction efficiency (*)	(* = 10^(-1/m) - 1) 1.00319
M	-3.3143
B	38.80329
R Value	0.9931
R^2 Value	0.98624
Start normalising from cycle	1
Noise Slope Correction	No
No Template Control Threshold	% 0
Reaction Efficiency Threshold	Disabled
Normalisation Method	Dynamic Tube Normalisation
Digital Filter	Light

Quantitation data for Cycling A.Green

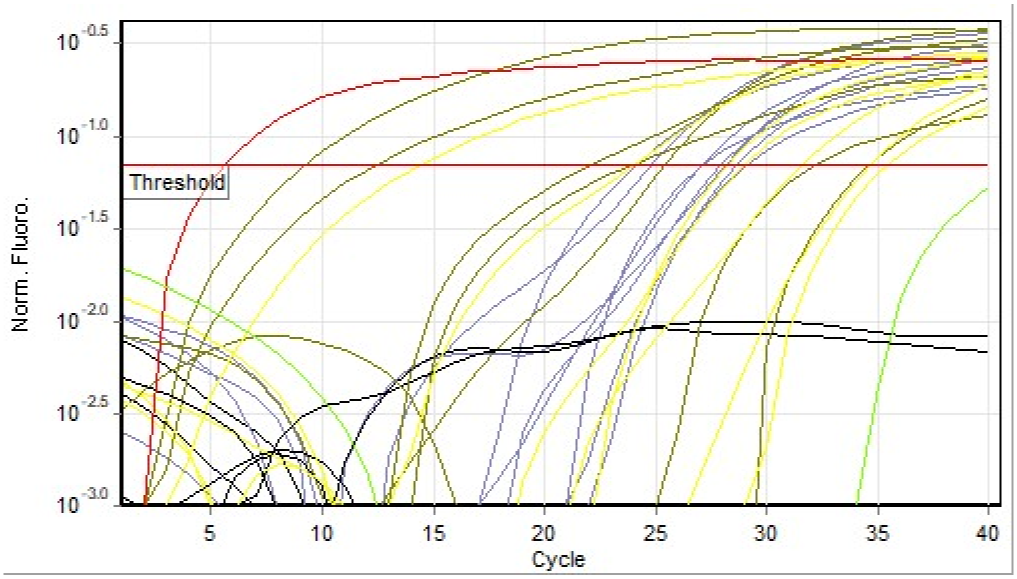

Standard Curve

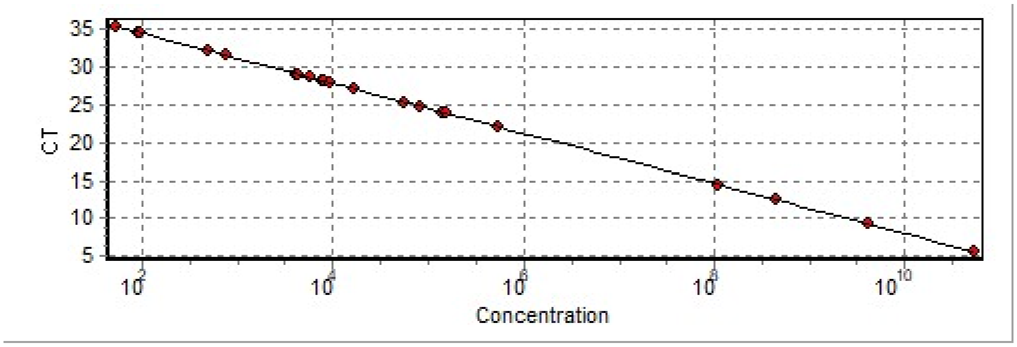

No.	Color	Name	Type	Ct	Ct Comment	Given Conc (Copies)	Calc Conc (Copies)
1		TrV_HD_12dpi	Unknown				
2		TrV_HD_12dpi	Unknown	27.11			16831.4205084
3		TrV_HD_12dpi	Unknown				
4		TrV_HD_12dpi	Unknown	27.09			17083.8082364
5		TrV_HD_12dpi	Unknown	28.63			5849.15120303
6		TrV_HD_12dpi	Unknown	29.11			4216.73626414
7		TrV_HD_12dpi	Unknown	27.93			9517.36702885

(Continued on next page)...

No.	Color	Name	Type	Ct	Ct Comment	Given Conc (Copies)	Calc Conc (Copies)
8		TrV_HD_12dpi	Unknown	24.06			140645.806075
9		TrV_HD_12dpi	Unknown	24.79			84284.9566268
10		TrV_HD_12dpi	Unknown	28.14			8265.88110988
11		TrV_HD_14dpi	Unknown				
12		TrV_HD_14dpi	Unknown	9.23			4190202824.90
13		TrV_HD_14dpi	Unknown	32.20			491.449631589
14		TrV_HD_14dpi	Unknown	23.90			156736.354684
15		TrV_HD_14dpi	Unknown	12.42			457286785.088
16		TrV_HD_14dpi	Unknown				
17		TrV_HD_14dpi	Unknown	25.38			55997.9190645
18		TrV_HD_14dpi	Unknown				
19		TrV_HD_14dpi	Unknown	22.10			546060.860489
20		TrV_HD_14dpi	Unknown	34.59			93.5258709341
21		TrV_HD_16dpi	Unknown	31.57			759.693444064
22		TrV_HD_16dpi	Unknown	14.44			112033639.177
23		TrV_HD_16dpi	Unknown	24.06			140181.389454
24		TrV_HD_16dpi	Unknown	28.21			7838.20395436
25		TrV_HD_16dpi	Unknown				
26		TrV_HD_16dpi	Unknown	34.61			92.1441660035
27		TrV_HD_16dpi	Unknown				
28		TrV_HD_16dpi	Unknown				
29		TrV_HD_16dpi	Unknown	35.40			53.2134121433
30		TrV_HD_16dpi	Unknown	29.05			4389.27817543
33		positive TrV	Positive Control	5.56			53480244176.5
37		negative TrV	NTC				

Legend:
NEG (NTC) - Sample cancelled due to NTC Threshold.
NEG (R. Eff) - Sample cancelled as efficiency less than reaction efficiency threshold.

This report was generated by Rotor-Gene Q Series Software 2.3.5 (Build 1)
Copyright © 2013 QIAGEN GmbH. All Rights Reserved.

QIAGEN

www.qiagen.com

Melt Report

Experiment Information

Run Name	qPCR_TrV_HD_12_14_16dpi
Run Start	13/04/2026 09:42:04 a. m.
Run Finish	13/04/2026 11:41:23 a. m.
Operator	Ever
Notes	
Run On Software Version	Rotor-Gene Q Software 2.3.5.1
Run Signature	The Run Signature is valid.
Gain Green	9.33
Machine Serial No.	0317130

Melt Information

Digital Filter	Light
Imported Analysis Settings	
Sample Page	Page 1
Temp. Threshold	0°C
Threshold	0.

Profile

Cycle	Cycle Point
Hold 1	Hold @ 95°C, 10min 0s
Cycling (40 repeats)	Step 1: Hold @ 95°C, 30s
	Step 2: Hold @ 58°C, 30s
	Step 3: Hold @ 72°C, 30s, acquiring to Cycling A([Green][1][1])
Hold 2	Hold @ 72°C, 10min 0s
Melt	Ramp from 60°C to 95°C
	Hold for 90s on the 1st step
	Hold for 5s on next steps,Melt A([Green][1][1])

Melt data for Melt A.Green

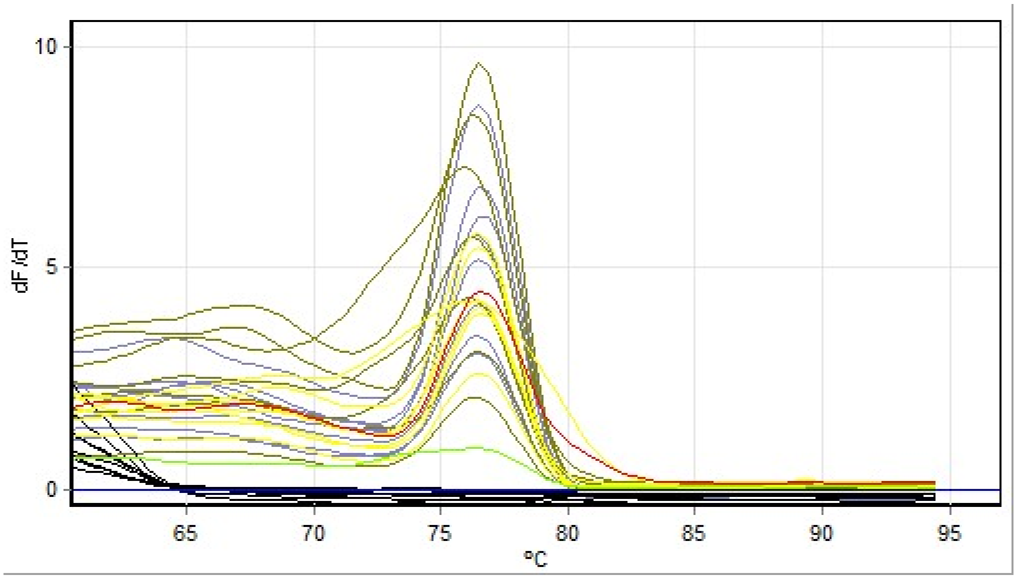

No.	Color	Name	Genotype	Peak 1	Peak 2	Peak 3	Peak 4
1		TrV_HD_12dpi					
2		TrV_HD_12dpi		62.0	76.5	81.7	85.5
3		TrV_HD_12dpi					
4		TrV_HD_12dpi		64.3	76.5	83.2	93.5
5		TrV_HD_12dpi		62.0	66.5	76.5	83.5
6		TrV_HD_12dpi		62.2	66.0	76.5	83.3

(Continued on next page)...

No.	Color	Name	Genotype	Peak 1	Peak 2	Peak 3	Peak 4
7		TrV_HD_12dpi		63.2	76.5	82.0	85.2
8		TrV_HD_12dpi		66.0	76.7	81.8	85.2
9		TrV_HD_12dpi		64.2	76.5	83.5	91.0
10		TrV_HD_12dpi		65.2	76.5		
11		TrV_HD_14dpi					
12		TrV_HD_14dpi		65.0	76.0	87.7	
13		TrV_HD_14dpi		64.8	76.3	83.2	87.5
14		TrV_HD_14dpi		63.0	76.2	84.5	88.5
15		TrV_HD_14dpi		65.0	76.3		
16		TrV_HD_14dpi		68.0	74.0		
17		TrV_HD_14dpi		62.5	67.0	76.5	81.7
18		TrV_HD_14dpi					
19		TrV_HD_14dpi		67.2	76.3	81.5	87.7
20		TrV_HD_14dpi		67.2	76.5	87.8	
21		TrV_HD_16dpi		64.0	76.5	91.0	
22		TrV_HD_16dpi		68.5	76.2	89.3	
23		TrV_HD_16dpi		68.5	76.5	81.7	87.0
24		TrV_HD_16dpi		61.5	67.0	76.5	82.0
25		TrV_HD_16dpi					
26		TrV_HD_16dpi		64.8	76.5		
27		TrV_HD_16dpi		70.5			
28		TrV_HD_16dpi					
29		TrV_HD_16dpi		61.5	66.8	76.5	83.3
30		TrV_HD_16dpi		76.5			
33		positive TrV		62.0	67.2	76.5	89.5
37		negative TrV		62.0	67.3	76.2	86.0

Bin Name	Temperature	Sample No.	Sample Name	Peak

This report was generated by Rotor-Gene Q Series Software 2.3.5 (Build 1)
Copyright © 2013 QIAGEN GmbH. All Rights Reserved.
