# Supplementary material for: Improved Purification and Quantitative Kinetics Monitoring of Triatoma virus Infection of Rhodnius prolixus
Source: Viruses. 2026 Sep 2;18(9):962. doi: 10.3390/v18090962 (PMC13611934; doi:10.3390/v18090962)
Supplement: Supplementary file 1 [file viruses-18-00962-s001.zip › Supplementary data/qPCR_Reports/Reports_S5/Report_S5.0_Quant_and_Melt_report_TrVgp1_LowDose_1_3_5dpi.pdf]

www.qiagen.com

Quantitation Report

Experiment Information

Run Name	qPCR_TrV_LW_1_3_5dpi
Run Start	15/10/2025 11:45:22 a. m.
Run Finish	15/10/2025 01:44:07 p. m.
Operator	Ever
Notes	
Run On Software Version	Rotor-Gene Q Software 2.3.5.1
Run Signature	The Run Signature is valid.
Gain Green	9.67
Machine Serial No.	0317130

Quantitation Information

Threshold	0.0697
Left Threshold	1.000
Standard Curve Imported	No
Standard Curve (1)	conc= 10^(-0.302*CT + 11.708)
Standard Curve (2)	CT = -3.314*log(conc) + 38.803
Reaction efficiency (*)	(* = 10^(-1/m) - 1) 1.00319
M	-3.3143
B	38.80329
R Value	0.9931
R^2 Value	0.98624
Start normalising from cycle	1
Noise Slope Correction	No
No Template Control Threshold	% 0
Reaction Efficiency Threshold	Disabled
Normalisation Method	Dynamic Tube Normalisation
Digital Filter	Light

Quantitation data for Cycling A.Green

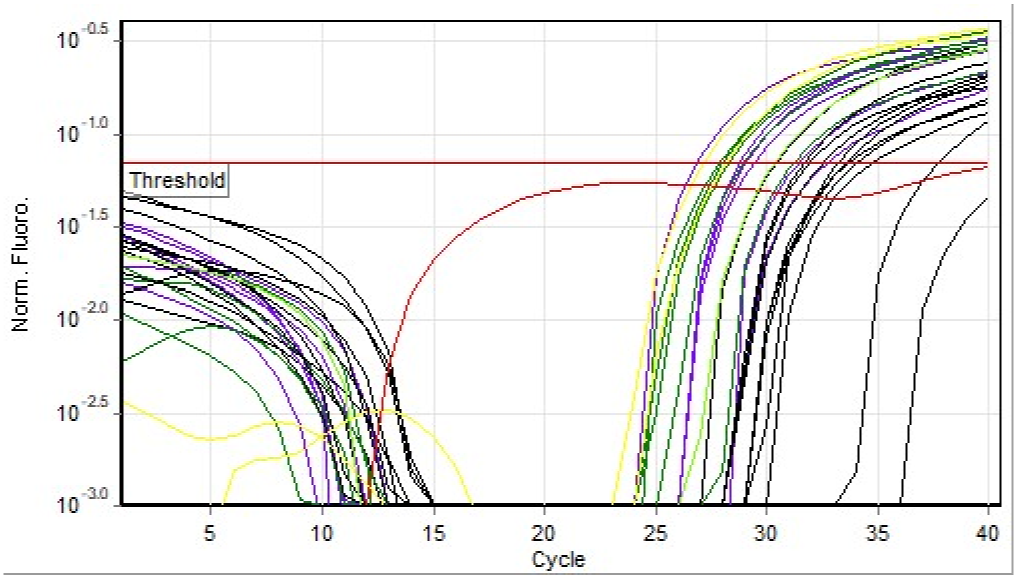

Standard Curve

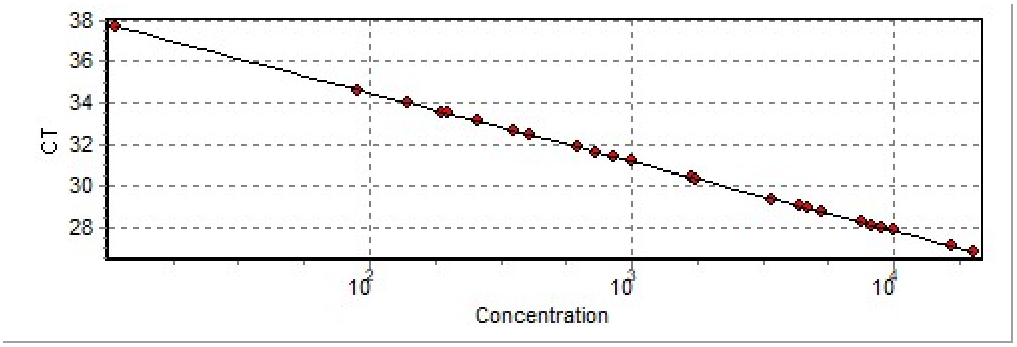

No.	Color	Name	Type	Ct	Ct Comment	Given Conc (copies/ul)	Calc Conc (copies/ul)
1		TrV_LW_1dpi	Unknown	31.41			853.106143438
2		TrV_LW_1dpi	Unknown	29.40			3434.93101539
3		TrV_LW_1dpi	Unknown	32.66			356.276004825
4		TrV_LW_1dpi	Unknown	28.78			5295.54042818
5		TrV_LW_1dpi	Unknown	30.41			1703.00580612
6		TrV_LW_1dpi	Unknown	26.85			20210.3153539
7		TrV_LW_1dpi	Unknown	29.04			4404.73016306

(Continued on next page)...

No.	Color	Name	Type	Ct	Ct Comment	Given Conc (copies/ul)	Calc Conc (copies/ul)
8		TrV_LW_1dpi	Unknown	33.13			257.216367542
9		TrV_LW_3dpi	Unknown	28.94			4719.59468784
10		TrV_LW_3dpi	Unknown	27.87			9925.25044602
11		TrV_LW_3dpi	Unknown	28.01			9010.02529427
12		TrV_LW_3dpi	Unknown	31.18			998.442563801
13		TrV_LW_3dpi	Unknown	37.69			10.8037354438
14		TrV_LW_3dpi	Unknown	31.86			623.078245019
15		TrV_LW_3dpi	Unknown	28.26			7611.46540937
16		TrV_LW_3dpi	Unknown				
17		TrV_LW_5dpi	Unknown	33.99			141.470073598
18		TrV_LW_5dpi	Unknown	32.47			406.839556025
19		TrV_LW_5dpi	Unknown	33.49			200.625941790
20		TrV_LW_5dpi	Unknown	31.64			726.368244437
21		TrV_LW_5dpi	Unknown	33.58			188.561966464
22		TrV_LW_5dpi	Unknown	27.13			16641.1392822
23		TrV_LW_5dpi	Unknown	28.14			8253.92879533
24		TrV_LW_5dpi	Unknown	34.63			90.6328658929
25		positive TrV	Positive Control				
28		negative TrV	NTC	30.36			1762.82347908

Legend:
NEG (NTC) - Sample cancelled due to NTC Threshold.
NEG (R. Eff) - Sample cancelled as efficiency less than reaction efficiency threshold.

This report was generated by Rotor-Gene Q Series Software 2.3.5 (Build 1)
Copyright © 2013 QIAGEN GmbH. All Rights Reserved.

QIAGEN

www.qiagen.com

Melt Report

Experiment Information

Run Name	qPCR_TrV_LW_1_3_5dpi
Run Start	15/10/2025 11:45:22 a. m.
Run Finish	15/10/2025 01:44:07 p. m.
Operator	Ever
Notes	
Run On Software Version	Rotor-Gene Q Software 2.3.5.1
Run Signature	The Run Signature is valid.
Gain Green	9.67
Machine Serial No.	0317130

Melt Information

Digital Filter	Light
Imported Analysis Settings	
Sample Page	Page 1
Temp. Threshold	0°C
Threshold	0.

Profile

Cycle	Cycle Point
Hold 1	Hold @ 95°C, 10min 0s
Cycling (40 repeats)	Step 1: Hold @ 95°C, 30s
	Step 2: Hold @ 58°C, 30s
	Step 3: Hold @ 72°C, 30s, acquiring to Cycling A([Green][1][1])
Hold 2	Hold @ 72°C, 10min 0s
Melt	Ramp from 60°C to 95°C
	Hold for 90s on the 1st step
	Hold for 5s on next steps,Melt A([Green][1][1])

Melt data for Melt A.Green

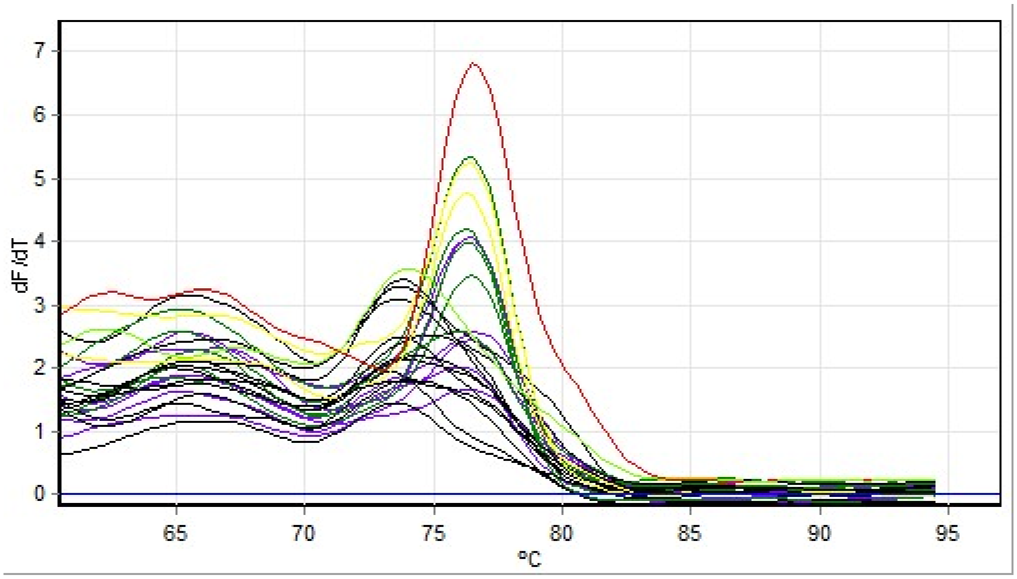

No.	Color	Name	Genotype	Peak 1	Peak 2	Peak 3	Peak 4
1		TrV_LW_1dpi		66.0	76.3	86.5	93.3
2		TrV_LW_1dpi		65.5	76.7	84.3	93.0
3		TrV_LW_1dpi		65.3	76.0	86.0	93.0
4		TrV_LW_1dpi		65.5	76.5		
5		TrV_LW_1dpi		65.5	73.7		
6		TrV_LW_1dpi		65.0	66.5	76.5	86.5

(Continued on next page)...

No.	Color	Name	Genotype	Peak 1	Peak 2	Peak 3	Peak 4
7		TrV_LW_1dpi		65.5	76.3		
8		TrV_LW_1dpi		64.8	74.0	84.8	
9		TrV_LW_3dpi		65.3	76.5	85.0	
10		TrV_LW_3dpi		65.2	76.5	85.7	92.5
11		TrV_LW_3dpi		65.2	76.2	84.8	92.8
12		TrV_LW_3dpi		64.5	76.0	86.0	92.3
13		TrV_LW_3dpi		66.0	73.3		
14		TrV_LW_3dpi		65.5	75.0	86.8	91.5
15		TrV_LW_3dpi		65.8	76.3		
16		TrV_LW_3dpi		66.5	73.5	85.7	
17		TrV_LW_5dpi		65.2	73.7	85.3	88.3
18		TrV_LW_5dpi		66.3	73.5	86.5	
19		TrV_LW_5dpi		65.5	73.8	87.5	
20		TrV_LW_5dpi		66.2	74.2	87.0	92.2
21		TrV_LW_5dpi		65.0	74.0		
22		TrV_LW_5dpi		65.8	76.3	87.2	
23		TrV_LW_5dpi		66.2	76.3	86.0	90.5
24		TrV_LW_5dpi		65.8	73.5	86.5	
25		positive TrV		62.5	66.0	76.5	89.5
28		negative TrV		62.2	67.5	74.0	85.2

Bin Name	Temperature	Sample No.	Sample Name	Peak

This report was generated by Rotor-Gene Q Series Software 2.3.5 (Build 1)
Copyright © 2013 QIAGEN GmbH. All Rights Reserved.
