# Supplementary material for: Improved Purification and Quantitative Kinetics Monitoring of Triatoma virus Infection of Rhodnius prolixus
Source: Viruses. 2026 Sep 2;18(9):962. doi: 10.3390/v18090962 (PMC13611934; doi:10.3390/v18090962)
Supplement: Supplementary file 1 [file viruses-18-00962-s001.zip › Supplementary data/qPCR_Reports/Reports_S5/Report_S5.1_Quant_and_Melt_report_TrVgp1_LowDose_7_9_12dpi.pdf]

QIAGEN

www.qiagen.com

Quantitation Report

Experiment Information

Run Name	qPCR_TrV_LW_7_9_12dpi
Run Start	21/10/2025 07:32:00 p. m.
Run Finish	21/10/2025 09:31:14 p. m.
Operator	Ever
Notes	
Run On Software Version	Rotor-Gene Q Software 2.3.5.1
Run Signature	The Run Signature is valid.
Gain Green	9.33
Machine Serial No.	0317130

Quantitation Information

Threshold	0.0697
Left Threshold	1.000
Standard Curve Imported	No
Standard Curve (1)	conc= 10^(-0.302*CT + 11.708)
Standard Curve (2)	CT = -3.314*log(conc) + 38.803
Reaction efficiency (*)	(* = 10^(-1/m) - 1) 1.00319
M	-3.3143
B	38.80329
R Value	0.9931
R^2 Value	0.98624
Start normalising from cycle	1
Noise Slope Correction	No
No Template Control Threshold	% 0
Reaction Efficiency Threshold	Disabled
Normalisation Method	Dynamic Tube Normalisation
Digital Filter	Light

Quantitation data for Cycling A.Green

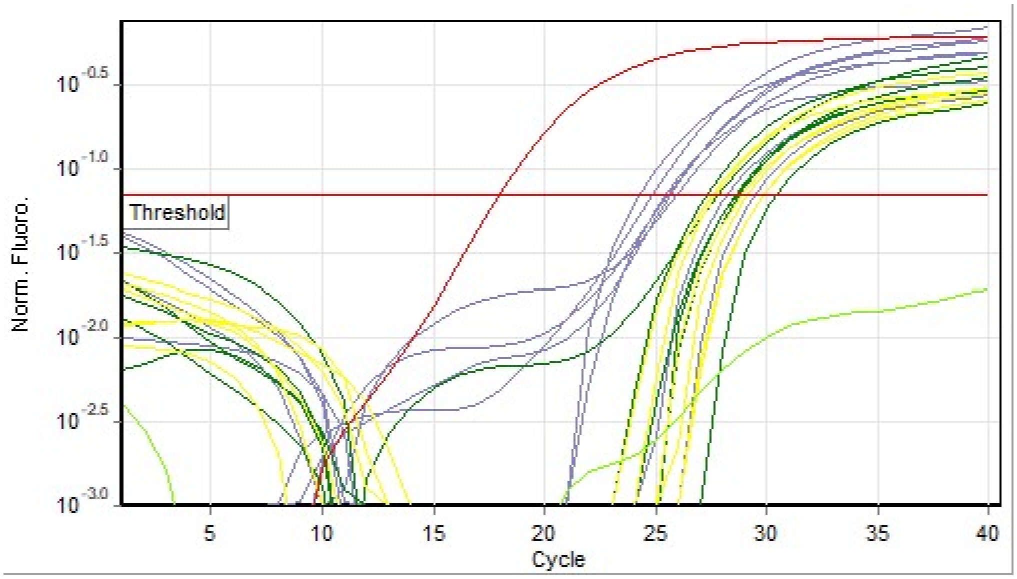

Standard Curve

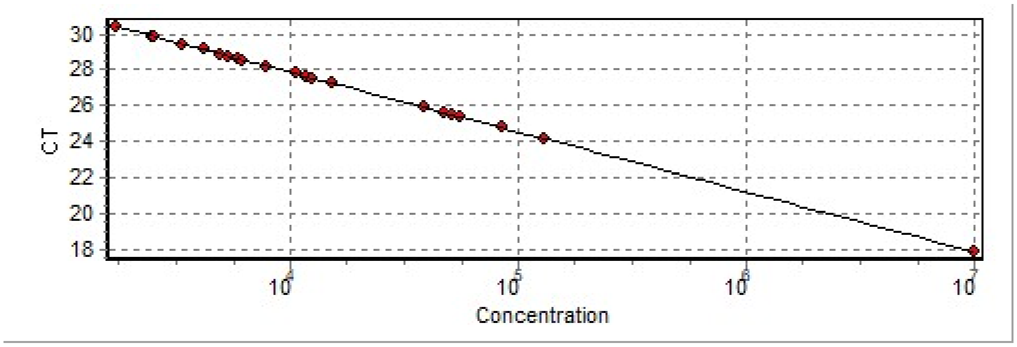

No.	Color	Name	Type	Ct	Ct Comment	Given Conc (copies/ul)	Calc Conc (copies/ul)
A1		TrV_LW_7dpi	Unknown	24.79			84669.1409733
A2		TrV_LW_7dpi	Unknown	25.51			51192.7936317
A3		TrV_LW_7dpi	Unknown	25.92			38447.5078198
A4		TrV_LW_7dpi	Unknown	25.64			46984.1665678
A5		TrV_LW_7dpi	Unknown	29.44			3354.23670215
A6		TrV_LW_7dpi	Unknown	28.20			7901.65108517
A7		TrV_LW_7dpi	Unknown	25.39			55674.7329799

(Continued on next page)...

No.	Color	Name	Type	Ct	Ct Comment	Given Conc (copies/ul)	Calc Conc (copies/ul)
A8		TrV_LW_7dpi	Unknown	24.18			129550.941942
B1		TrV_LW_9dpi	Unknown	30.40			1720.69921370
B4		TrV_LW_9dpi	Unknown	27.25			15342.6662873
B5		TrV_LW_9dpi	Unknown	27.79			10514.5128159
B6		TrV_LW_9dpi	Unknown	28.62			5927.04947822
B7		TrV_LW_9dpi	Unknown	28.75			5381.61797551
B8		TrV_LW_9dpi	Unknown	28.56			6177.23152538
C1		TrV_LW_12dpi	Unknown	29.86			2490.66521769
C2		TrV_LW_12dpi	Unknown	27.64			11632.9517830
C4		TrV_LW_12dpi	Unknown	27.53			12575.8027957
C5		TrV_LW_12dpi	Unknown	28.87			4957.60067393
C6		TrV_LW_12dpi	Unknown	29.12			4186.33768117
C8		TrV_LW_12dpi	Unknown	29.83			2551.11152779
E4		positive TrV	Positive Control	17.91			10034034.4654
F1		neg	NTC				

Legend:
NEG (NTC) - Sample cancelled due to NTC Threshold.
NEG (R. Eff) - Sample cancelled as efficiency less than reaction efficiency threshold.

This report was generated by Rotor-Gene Q Series Software 2.3.5 (Build 1)
Copyright © 2013 QIAGEN GmbH. All Rights Reserved.

www.qiagen.com

Melt Report

Experiment Information

Run Name	qPCR_TrV_LW_7_9_12dpi
Run Start	21/10/2025 07:32:00 p. m.
Run Finish	21/10/2025 09:31:14 p. m.
Operator	Ever
Notes	
Run On Software Version	Rotor-Gene Q Software 2.3.5.1
Run Signature	The Run Signature is valid.
Gain Green	9.33
Machine Serial No.	0317130

Melt Information

Digital Filter	Light
Imported Analysis Settings	
Sample Page	Page 1
Temp. Threshold	0°C
Threshold	0.

Profile

Cycle	Cycle Point
Hold 1	Hold @ 95°C, 10min 0s
Cycling (40 repeats)	Step 1: Hold @ 95°C, 30s
	Step 2: Hold @ 58°C, 30s
	Step 3: Hold @ 72°C, 30s, acquiring to Cycling A([Green][1][1])
Hold 2	Hold @ 72°C, 10min 0s
Melt	Ramp from 60°C to 95°C
	Hold for 90s on the 1st step
	Hold for 5s on next steps,Melt A([Green][1][1])

Melt data for Melt A.Green

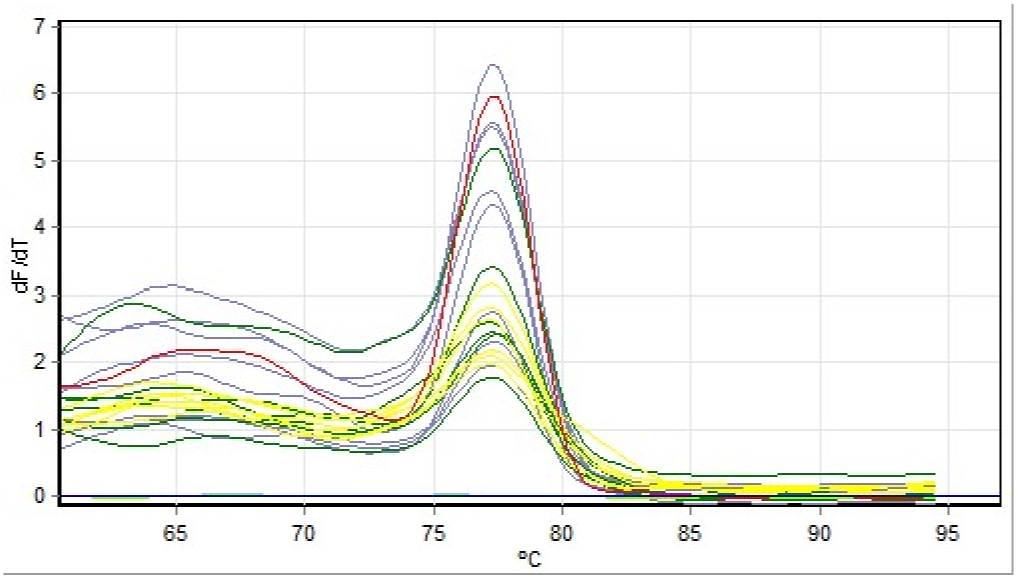

No.	Color	Name	Genotype	Peak 1	Peak 2	Peak 3	Peak 4
A1		TrV_LW_7dpi		65.0	77.3		
A2		TrV_LW_7dpi		64.0	67.7	77.3	92.0
A3		TrV_LW_7dpi		65.3	77.2	86.7	90.5
A4		TrV_LW_7dpi		64.8	77.3	89.5	
A5		TrV_LW_7dpi		64.0	69.0	77.2	88.2
A6		TrV_LW_7dpi		65.3	77.3	84.3	88.3

(Continued on next page)...

No.	Color	Name	Genotype	Peak 1	Peak 2	Peak 3	Peak 4
A7		TrV_LW_7dpi		63.8	66.2	77.2	87.8
A8		TrV_LW_7dpi		65.3	77.3	87.2	93.0
B1		TrV_LW_9dpi		66.5	77.3		
B4		TrV_LW_9dpi		65.0	70.3	77.3	85.3
B5		TrV_LW_9dpi		63.5	67.5	77.3	89.7
B6		TrV_LW_9dpi		63.5	77.3	87.8	
B7		TrV_LW_9dpi		63.2	67.2	77.2	89.5
B8		TrV_LW_9dpi		65.5	77.5		
C1		TrV_LW_12dpi		63.5	66.0	77.2	85.7
C2		TrV_LW_12dpi		64.7	77.0	91.0	
C4		TrV_LW_12dpi		64.0	77.2	90.5	
C5		TrV_LW_12dpi		64.7	69.2	77.0	84.0
C6		TrV_LW_12dpi		65.0	77.2	88.5	
C8		TrV_LW_12dpi		66.3	77.2	86.3	
E4		positive TrV		65.5	77.3		
F1		neg		67.2	71.8	75.7	80.0

Bin Name	Temperature	Sample No.	Sample Name	Peak

This report was generated by Rotor-Gene Q Series Software 2.3.5 (Build 1)
Copyright © 2013 QIAGEN GmbH. All Rights Reserved.
