# Supplementary material for: Improved Purification and Quantitative Kinetics Monitoring of Triatoma virus Infection of Rhodnius prolixus
Source: Viruses. 2026 Sep 2;18(9):962. doi: 10.3390/v18090962 (PMC13611934; doi:10.3390/v18090962)
Supplement: Supplementary file 1 [file viruses-18-00962-s001.zip › Supplementary data/qPCR_Reports/Reports_S6/Report_S6.0_Quant_and_Melt_reports_controls_1_3_5_7_dpi.pdf]

QIAGEN

www.qiagen.com

Quantitation Report

Experiment Information

Run Name	Controls_1_3_5_7_dpi
Run Start	08/08/2026 12:26:33 p. m.
Run Finish	08/08/2026 02:25:29 p. m.
Operator	Ever
Notes	
Run On Software Version	Rotor-Gene Q Software 2.3.5.1
Run Signature	No Run Signature found. Run file contents not guaranteed.
Gain Green	9.33
Machine Serial No.	0317130

Quantitation Information

Threshold	0.0697
Left Threshold	1.000
Standard Curve Imported	No
Standard Curve (1)	conc= 10^(-0.302*CT + 11.708)
Standard Curve (2)	CT = -3.314*log(conc) + 38.803
Reaction efficiency (*)	(* = 10^(-1/m) - 1) 1.00319
M	-3.3143
B	38.80329
R Value	0.9931
R^2 Value	0.98624
Start normalising from cycle	1
Noise Slope Correction	No
No Template Control Threshold	% 0
Reaction Efficiency Threshold	Disabled
Normalisation Method	Dynamic Tube Normalisation
Digital Filter	Light

Quantitation data for Cycling A.Green

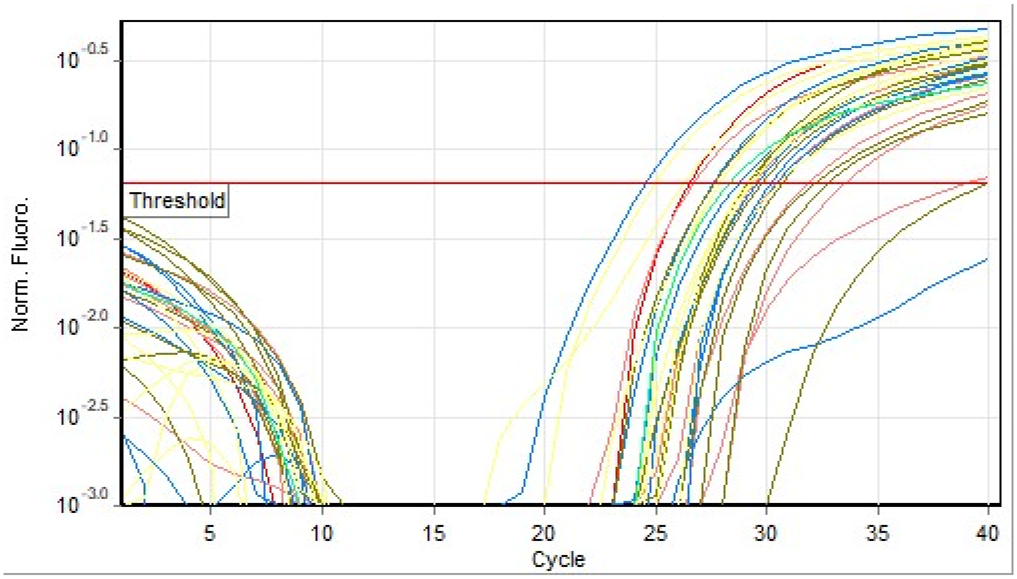

Standard Curve

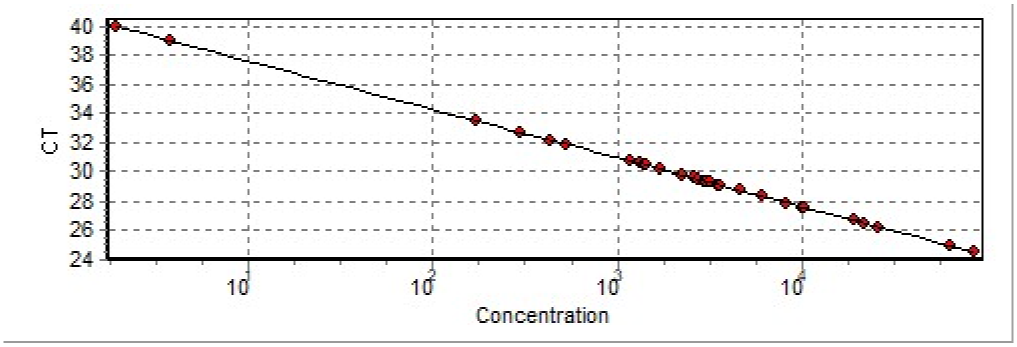

No.	Color	Name	Type	Ct	Ct Comment	Given Conc (Copies)	Calc Conc (Copies)
A2		positive TrV	Positive Control	26.49			21892.5688361
C6		Control_1	Unknown	33.47			172.063039846
C7		Control_1	Unknown	31.84			532.783390553
D2		Control_1	Unknown	26.68			19160.9112338
D5		Control_1	Unknown	30.43			1416.40557818
D6		Control_1	Unknown	29.46			2779.14197605
D7		Control_1	Unknown	38.99			3.71214295811

(Continued on next page)...

No.	Color	Name	Type	Ct	Ct Comment	Given Conc (Copies)	Calc Conc (Copies)
D8		Control_3	Unknown	27.58			10278.9299514
E1		Control_3	Unknown	29.56			2595.42022278
E2		Control_3	Unknown				
E6		Control_3	Unknown	30.16			1712.95499871
E7		Control_3	Unknown	24.51			86363.8386317
E8		Control_3	Unknown	30.45			1397.22055475
F1		Control_3	Unknown	28.72			4656.66005040
F2		Control_5	Unknown	30.70			1178.96495866
F3		Control_5	Unknown	32.14			432.072917000
F4		Control_5	Unknown	39.95			1.90840503895
F7		Control_5	Unknown	27.61			10037.5591098
F8		Control_5	Unknown	29.34			3032.35385192
G1		Control_5	Unknown	29.12			3537.72370766
G2		Control_5	Unknown	32.68			297.134471853
G3		Control_5	Unknown	29.78			2226.95959506
G4		Control_7	Unknown	29.38			2944.67372128
G5		Control_7	Unknown	27.89			8291.27088006
G6		Control_7	Unknown	29.28			3159.64000120
H1		Control_7	Unknown	24.93			64682.8936901
H2		Control_7	Unknown	26.23			26274.5186627
H3		Control_7	Unknown	27.88			8325.60591689
H4		Control_7	Unknown	29.08			3624.29197358
H5		Control_7	Unknown	30.53			1320.04034737
H7		negative TrV	NTC	28.33			6104.22327529

Legend:
NEG (NTC) - Sample cancelled due to NTC Threshold.
NEG (R. Eff) - Sample cancelled as efficiency less than reaction efficiency threshold.

This report was generated by Rotor Gene Q Series Software 2.3.5 (Build 1)
Copyright © 2013 QIAGEN GmbH. All Rights Reserved.

www.qiagen.com

Melt Report

Experiment Information

Run Name	Controls_1_3_5_7_dpi
Run Start	08/08/2026 12:26:33 p. m.
Run Finish	08/08/2026 02:25:29 p. m.
Operator	Ever
Notes	
Run On Software Version	Rotor-Gene Q Software 2.3.5.1
Run Signature	No Run Signature found. Run file contents not guaranteed.
Gain Green	9.33
Machine Serial No.	0317130

Melt Information

Digital Filter	Light
Imported Analysis Settings	
Sample Page	Page 1
Temp. Threshold	0°C
Threshold	0.

Profile

Cycle	Cycle Point
Hold 1	Hold @ 95°C, 10min 0s
Cycling (40 repeats)	Step 1: Hold @ 95°C, 30s
	Step 2: Hold @ 58°C, 30s
	Step 3: Hold @ 72°C, 30s, acquiring to Cycling A([Green][1][1])
Hold 2	Hold @ 72°C, 10min 0s
Melt	Ramp from 60°C to 95°C
	Hold for 90s on the 1st step
	Hold for 5s on next steps,Melt A([Green][1][1])

Melt data for Melt A.Green

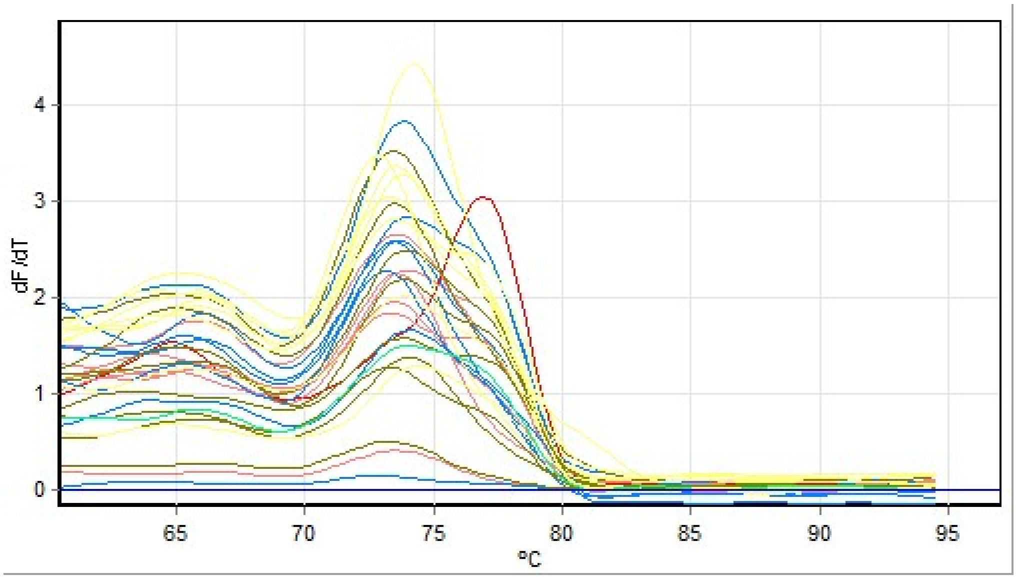

No.	Color	Name	Genotype	Peak 1	Peak 2	Peak 3	Peak 4
A2		positive TrV		64.8	77.0	82.8	85.5
C6		Control_1		66.3	73.5	91.5	
C7		Control_1		65.8	73.5	87.7	92.7
D2		Control_1		64.0	74.0	84.3	88.2
D5		Control_1		64.3	73.3	76.2	82.0
D6		Control_1		66.0	73.5		

(Continued on next page)...

No.	Color	Name	Genotype	Peak 1	Peak 2	Peak 3	Peak 4
D7		Control_1		66.5	73.5	82.8	
D8		Control_3		66.0	74.0		
E1		Control_3		65.3	73.7		
E2		Control_3		63.7	72.8	81.5	87.2
E6		Control_3		61.5	66.0	73.5	87.0
E7		Control_3		65.2	73.8	83.2	91.0
E8		Control_3		65.2	73.2		
F1		Control_3		64.0	66.0	74.0	87.3
F2		Control_5		63.5	73.7	84.7	90.5
F3		Control_5		66.0	74.0	85.3	90.3
F4		Control_5		66.5	73.3	85.7	93.5
F7		Control_5		65.0	74.0	82.5	88.0
F8		Control_5		64.7	73.5	86.3	91.5
G1		Control_5		65.7	73.8	84.0	89.5
G2		Control_5		64.7	73.3	84.5	89.3
G3		Control_5		65.0	73.5	84.2	89.0
G4		Control_7		65.5	73.8	91.0	
G5		Control_7		65.3	73.5	85.8	93.2
G6		Control_7		62.3	66.0	72.8	86.5
H1		Control_7		64.5	73.8	85.0	88.0
H2		Control_7		65.0	74.2	85.5	90.8
H3		Control_7		66.0	73.5	85.8	90.3
H4		Control_7		65.5	73.3	85.3	89.2
H5		Control_7		64.7	74.5	90.2	
H7		negative TrV		62.0	65.7	74.0	83.5

Bin Name	Temperature	Sample No.	Sample Name	Peak

This report was generated by Rotor-Gene Q Series Software 2.3.5 (Build 1)
Copyright © 2013 QIAGEN GmbH. All Rights Reserved.
