# Supplementary material for: Improved Purification and Quantitative Kinetics Monitoring of Triatoma virus Infection of Rhodnius prolixus
Source: Viruses. 2026 Sep 2;18(9):962. doi: 10.3390/v18090962 (PMC13611934; doi:10.3390/v18090962)
Supplement: Supplementary file 1 [file viruses-18-00962-s001.zip › Supplementary data/qPCR_Reports/Reports_S6/Report_S6.1_Quant_and_Melt_reports_controls_9_12_14_16_dpi.pdf]

QIAGEN

www.qiagen.com

Quantitation Report

Experiment Information

Run Name	Controls_9_12_14_16_dpi
Run Start	08/08/2026 03:19:36 p. m.
Run Finish	08/08/2026 05:18:53 p. m.
Operator	Ever
Notes	
Run On Software Version	Rotor-Gene Q Software 2.3.5.1
Run Signature	The Run Signature is valid.
Gain Green	9.33
Machine Serial No.	0317130

Quantitation Information

Threshold	0.0697
Left Threshold	1.000
Standard Curve Imported	No
Standard Curve (1)	conc= 10^(-0.302*CT + 11.708)
Standard Curve (2)	CT = -3.314*log(conc) + 38.803
Reaction efficiency (*)	(* = 10^(-1/m) - 1) 1.00319
M	-3.3143
B	38.80329
R Value	0.9931
R^2 Value	0.98624
Start normalising from cycle	1
Noise Slope Correction	No
No Template Control Threshold	% 0
Reaction Efficiency Threshold	Disabled
Normalisation Method	Dynamic Tube Normalisation
Digital Filter	Light

Quantitation data for Cycling A.Green

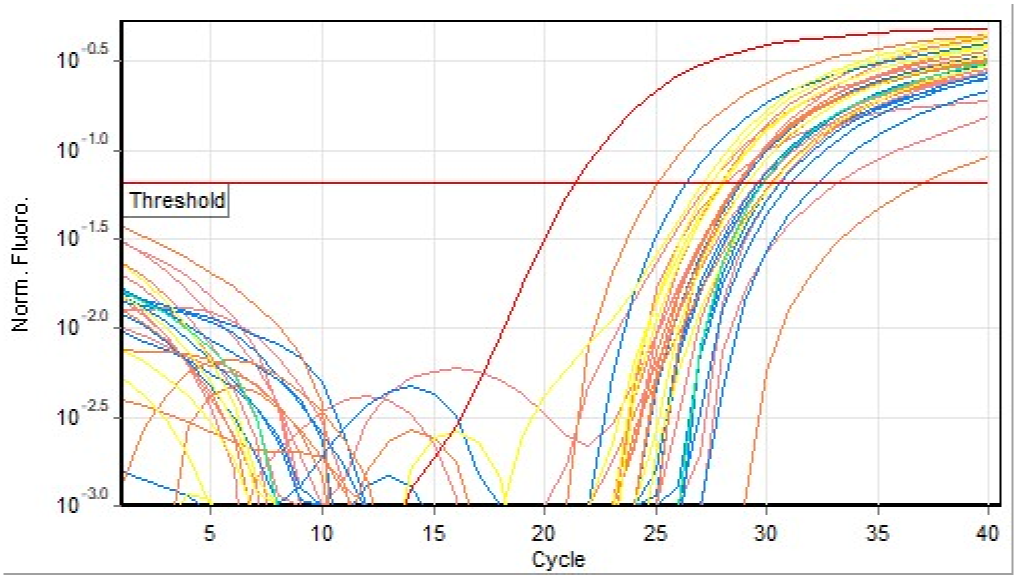

Standard Curve

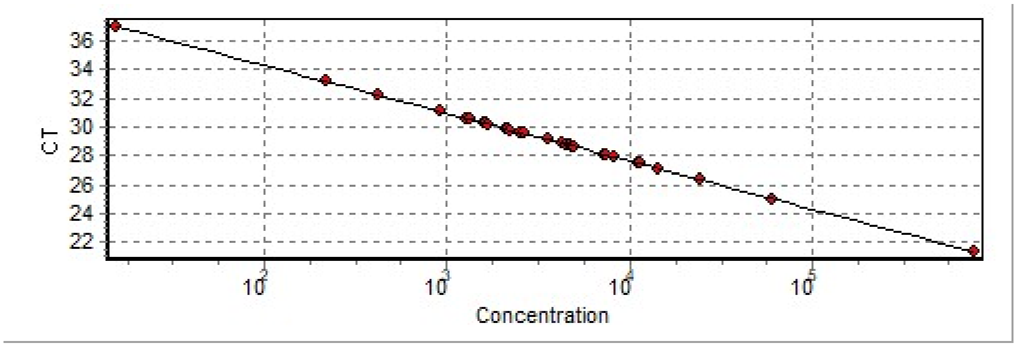

No.	Color	Name	Type	Ct	Ct Comment	Given Conc (Copies)	Calc Conc (Copies)
A1		Control_9	Unknown	27.90			8226.42393174
A2		Control_9	Unknown	33.14			216.777350513
A3		Control_9	Unknown	28.79			4447.87784590
A4		Control_9	Unknown	28.65			4903.53028428
A5		Control_9	Unknown	29.87			2100.90500664
A6		Control_9	Unknown	28.09			7213.36913166
A7		Control_9	Unknown	27.43			11411.8219044

(Continued on next page)...

No.	Color	Name	Type	Ct	Ct Comment	Given Conc (Copies)	Calc Conc (Copies)
A8		Control_9	Unknown	30.53			1322.49744453
B1		Control_9	Unknown	29.53			2654.53863759
B2		Control_9	Unknown	30.22			1644.28829905
B3		Contrrol_12	Unknown	31.08			905.911612685
B4		Contrrol_12	Unknown	32.20			416.728320417
B6		Contrrol_12	Unknown	29.59			2541.81662488
B7		Contrrol_12	Unknown	30.28			1579.02662700
B8		Contrrol_12	Unknown	30.60			1264.77007819
C1		Contrrol_12	Unknown	28.86			4233.55917367
C3		Contrrol_12	Unknown	26.33			24390.9848854
C4		Contrrol_12	Unknown	29.78			2234.33420666
C5		Control_14	Unknown	29.11			3539.18599387
C6		Control_14	Unknown	30.19			1673.76844109
C7		Control_14	Unknown	36.94			15.4586328122
D2		Control_14	Unknown	28.67			4829.12337458
D3		Control_14	Unknown	28.64			4913.67281569
D4		Control_14	Unknown	28.71			4697.24812097
D5		Control_14	Unknown	25.04			60095.6390998
D6		Control_16	Unknown	27.91			8157.02618614
D7		Control_16	Unknown	27.10			14344.7897611
D8		Control_16	Unknown	30.26			1598.06418168
E1		Control_16	Unknown	29.09			3592.23279099
E2		Control_16	Unknown	27.48			10990.6352470
E3		Control_16	Unknown	28.05			7420.48289802
E4		positive TrV	Positive Control	21.37			766155.424198
F2		negative TrV	NTC	29.82			2173.31828984

Legend:
NEG (NTC) - Sample cancelled due to NTC Threshold.
NEG (R. Eff) - Sample cancelled as efficiency less than reaction efficiency threshold.

This report was generated by Rotor-Gene Q Series Software 2.3.5 (Build 1)
Copyright © 2013 QIAGEN GmbH. All Rights Reserved.

www.qiagen.com

Melt Report

Experiment Information

Run Name	Controls_9_12_14_16_dpi
Run Start	08/08/2026 03:19:36 p. m.
Run Finish	08/08/2026 05:18:53 p. m.
Operator	Ever
Notes	
Run On Software Version	Rotor-Gene Q Software 2.3.5.1
Run Signature	The Run Signature is valid.
Gain Green	9.33
Machine Serial No.	0317130

Melt Information

Digital Filter	Light
Imported Analysis Settings	
Sample Page	Page 1
Temp. Threshold	0°C
Threshold	0.

Profile

Cycle	Cycle Point
Hold 1	Hold @ 95°C, 10min 0s
Cycling (40 repeats)	Step 1: Hold @ 95°C, 30s
	Step 2: Hold @ 58°C, 30s
	Step 3: Hold @ 72°C, 30s, acquiring to Cycling A([Green][1][1])
Hold 2	Hold @ 72°C, 10min 0s
Melt	Ramp from 60°C to 95°C
	Hold for 90s on the 1st step
	Hold for 5s on next steps,Melt A([Green][1][1])

Melt data for Melt A.Green

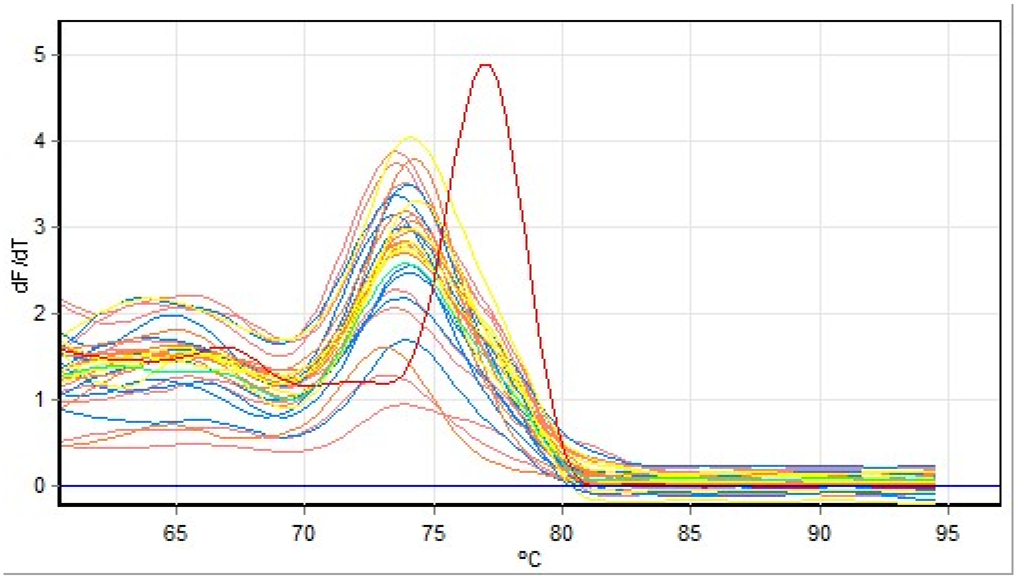

No.	Color	Name	Genotype	Peak 1	Peak 2	Peak 3	Peak 4
A1		Control_9		63.8	74.0	85.5	92.5
A2		Control_9		63.8	66.5	73.2	86.3
A3		Control_9		65.5	73.5		
A4		Control_9		65.2	73.5		
A5		Control_9		65.5	73.5		
A6		Control_9		65.7	73.7	86.5	91.8

(Continued on next page)...

No.	Color	Name	Genotype	Peak 1	Peak 2	Peak 3	Peak 4
A7		Control_9		64.0	74.2	88.5	93.0
A8		Control_9		62.0	66.3	73.5	82.5
B1		Control_9		64.0	74.0	85.8	91.0
B2		Control_9		63.0	73.8	87.5	92.2
B3		Contrrol_12		66.0	74.0		
B4		Contrrol_12		66.0	74.0	82.5	91.3
B6		Contrrol_12		64.8	74.0	85.3	93.0
B7		Contrrol_12		64.2	73.7	83.5	88.7
B8		Contrrol_12		63.8	73.5	90.5	
C1		Contrrol_12		65.2	73.8		
C3		Contrrol_12		64.8	74.0		
C4		Contrrol_12		63.5	73.5	84.5	87.8
C5		Control_14		64.7	73.7		
C6		Control_14		65.0	73.8	85.2	90.5
C7		Control_14		64.7	73.0	87.8	93.3
D2		Control_14		65.5	73.8	83.7	87.0
D3		Control_14		65.5	74.2	84.0	86.2
D4		Control_14		63.8	74.0	83.7	89.0
D5		Control_14		65.2	74.2	86.5	91.3
D6		Control_16		65.0	74.0	86.8	92.0
D7		Control_16		64.0	74.0	87.0	93.0
D8		Control_16		63.8	74.0	86.3	91.5
E1		Control_16		65.5	73.8	82.5	86.5
E2		Control_16		63.5	66.3	74.3	
E3		Control_16		65.7	74.0		
E4		positive TrV		66.8	71.8	77.0	83.2
F2		negative TrV		62.5	66.0	74.0	83.7

Bin Name	Temperature	Sample No.	Sample Name	Peak

This report was generated by Rotor-Gene Q Series Software 2.3.5 (Build 1)
Copyright © 2013 QIAGEN GmbH. All Rights Reserved.
