# Supplementary material for: Improved Purification and Quantitative Kinetics Monitoring of Triatoma virus Infection of Rhodnius prolixus
Source: Viruses. 2026 Sep 2;18(9):962. doi: 10.3390/v18090962 (PMC13611934; doi:10.3390/v18090962)
Supplement: Supplementary file 1 [file viruses-18-00962-s001.zip › Supplementary data/Supplementary_Figures.pdf]

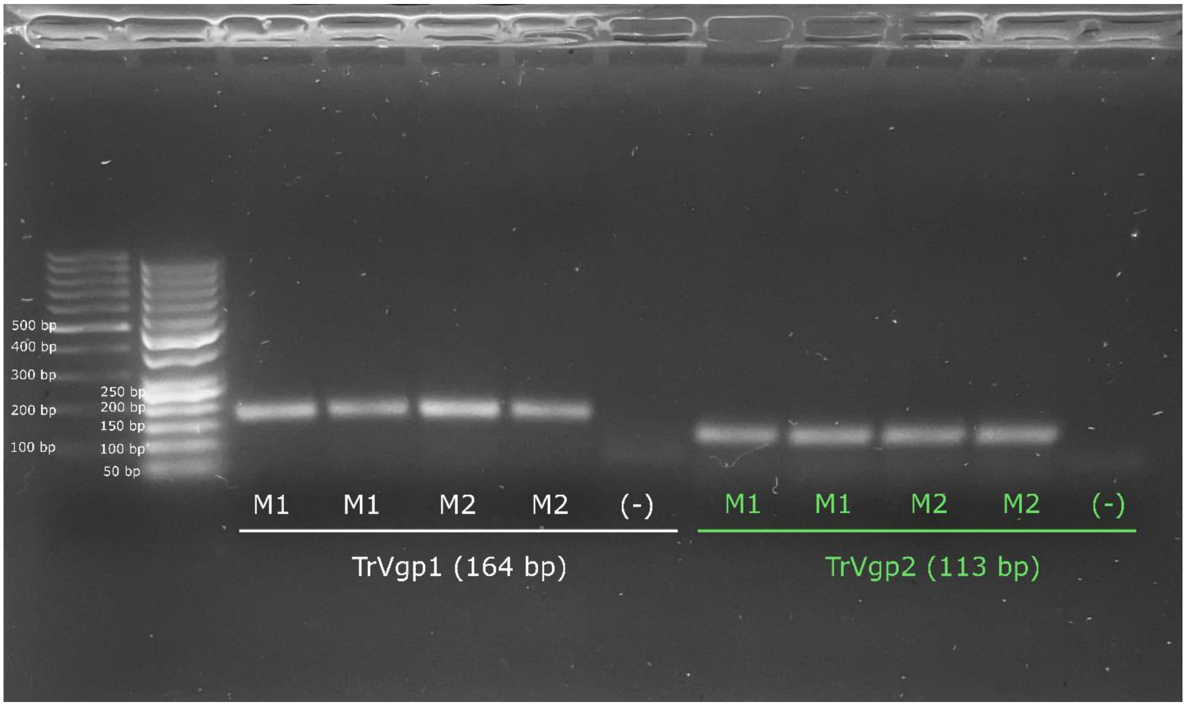

Figure S1. Duplicate verification of TrV end-point PCR products. Agarose gel electrophoresis confirming the specificity of the designed primer pairs. Biological samples (M1 and M2) evaluated in duplicate exhibit homogenous bands of 164 bp for the *TrVgp1* region and 113 bp for the *TrVgp2* region. Lanes labeled as (-) represent the negative control, validating the absence of contamination or primer-dimers in the reaction mixture. The reference molecular weight marker is visible on the left (50 to 500 bp).
