# Supplementary material for: Improved Purification and Quantitative Kinetics Monitoring of Triatoma virus Infection of Rhodnius prolixus
Source: Viruses. 2026 Sep 2;18(9):962. doi: 10.3390/v18090962 (PMC13611934; doi:10.3390/v18090962)
Supplement: Supplementary file 1 [file viruses-18-00962-s001.zip › Supplementary data/Supplementary_Tables.pdf]

Table S1 Operational parameters for the four TrV isolation and purification methods. Sample / Buffer indicates the initial starting mass of insect tissue and the specific volume and type of stabilization buffer used for mechanical homogenization. **Clarification** refers to the initial separation phase; Method 1 utilized high-speed ultracentrifugation (UC) to precipitate the virus directly, whereas Methods 2, 3, and 4 employed successive low-speed microcentrifugations (MC) to sediment heavy host tissue debris while retaining the virus-containing supernatant. **Gradient (UC)** defines the sucrose density matrix geometry (continuous or discontinuous) and the specific ultracentrifugation parameters utilized to isolate viral particles based on their buoyant density. **Fractions** indicates the volumetric size of the aliquots collected after density gradient separation. **Washing / Closure** comprises the final operational steps, involving an independent ultracentrifugation round to eliminate residual sucrose from the matrix, followed by the reconstitution of the purified viral pellet in TE buffer, or the direct collection of the enriched supernatant.

Process Step	Method 1 (Martí et al., 2008)	Method 2 (Susevich et al., 2017)	Method 3 (EGM)	Method 4 (Clarification Only)
**Sample / Buffer**	0.05 g / 10 mL NMT	0.05 g / 150 μL PBS	0.05 g / 300 μL NMT	0.05 g / 300 μL NMT
**Clarification**	UC: 151,637 *g*, 3 h at 4°C *(Retains pellet)*	3× MC: 13,000 *g*, 10 min *(Retains supernatant)*	3× MC: 15,000 *g*, 10 min, 25°C *(Retains supernatant)*	3× MC: 15,000 *g*, 10 min, 25°C *(Retains supernatant)*
**Gradient (UC)**	Continuous (10-30%, w/v) UC: 79,559 *g*, 3 h, 4°C	Continuous (10-30%, w/v) UC: 54,974 *g*, 3 h, 4°C	Discontinuous (20/30/60%, w/v) UC: 82,844 *g*, 2 h 50 min, 4°C	N/A *(No UC)*
**Fractions**	2 mL	2 mL	2 mL	N/A
**Washing / Closure**	UC: 44,979 *g*, 2 h, 4°C Resuspension: 1 mL TE.	N/A. *(Ready in fraction)*	UC: 44,000 *g*, 2 h, 4°C Resuspension: 1 mL TE.	N/A. *(Direct supernatant)*

Abbreviations: **EGM**, Everardo Gutiérrez Millán; **MC**, microcentrifugation; **UC**, ultracentrifugation; **N/A**, not applicable; **w/v**, weight/volume. *Buffers:* **NMT**, 0.01 M NaCl, 0.001 M $MgCl_2$, 0.04 M Tris-HCl (pH 7.4); **PBS**, phosphate-buffered saline; **TE**, 1 mM EDTA, 10 mM Tris-HCl (pH 7.4).

Table S2. Comparative analysis of *Triatoma virus* (TrV) purification efficiency and absolute quantification across different methods. Viral loads were determined via absolute qPCR targeting the *TrVgp1* gene, using a standardized technical template input of 100 ng of cDNA per reaction to define cycle threshold (Ct) values. For the density gradient methodologies (Susevich, EGM, and Martí), consecutive fractions from two independent purification replicas (_1 and _2) are shown. For the microcentrifuge method, individual tube extraction outcomes are detailed. Values represent total RNA and cDNA concentrations (ng/µL), raw Ct values, and the calculated absolute viral titters expressed as viral genome copies per microliter (copies/µL).

Method	Fraction	[] RNA ng/µl	[] cDNA ng/µl	Ct	Viral copies
Susevich	F1_1	11.2	246	13.38	2.34E+08
Susevich	F2_1	21.8	325	16.39	2.89E+07
Susevich	F3_1	12.3	347	11.46	8.90E+08
Susevich	F4_1	26	435	16.77	2.23E+07
Susevich	F1_2	11	407	12.09	5.76E+08
Susevich	F2_2	20	461	14.6	1.00E+08
Susevich	F3_2	12	412	14.47	1.10E+08
Susevich	F4_2	23	453	16.9	2.03E+07
EGM	F1_1	28.5	425	18.3	7.66E+06
EGM	F2_1	26.5	409	10.53	1.70E+09
EGM	F3_1	20.7	558	8.65	6.28E+09
EGM	F4_1	11.5	481	11.31	9.85E+08
EGM	F5_1	27.6	428	17.93	9.90E+06
EGM	F1_2	19.5	448	8.11	9.11E+09
EGM	F2_2	19.5	512	17.68	9.97E+06
EGM	F3_2	11.5	511	11.22	1.05E+09
EGM	F4_2	27.6	456	12.9	2.75E+08
EGM	F5_2	25.8	498	13.31	2.47E+08
Martí	F1_1	69.1	72.8	14.83	8.57E+07
Martí	F2_1	157	109	19.25	3.97E+06
Martí	F3_1	113	81.6	21.31	9.52E+05
Martí	F4_1	93.1	129	21.42	8.77E+05
Martí	F5_1	112	90	19.29	3.87E+06
Martí	F1_2	56.6	159	17.32	1.52E+07
Martí	F2_2	55	135	9.42	3.67E+09
Martí	F3_2	64	139	9.5	3.48E+09
Martí	F4_2	149	172	17.23	1.62E+07
Martí	F5_2	20.2	201	13.13	2.78E+08
Microcentrifuge	Tube1	63.1	675	19.03	4.62E+06
Microcentrifuge	Tube2	35.8	686	29.44	3.35E+03
Microcentrifuge	Tube3	18	663	31.74	6.78E+02
Microcentrifuge	Tube4	57.4	772	22.51	4.13E+05
Microcentrifuge	Tube5	29.5	768	31.12	1.04E+03
Microcentrifuge	Tube6	44.9	848	30.89	1.22E+03

Table S3.Experimental groups, inoculum compositions, and estimated viral doses per insect.

Experimental Group	Inoculum Composition	Viral Dose per Insect	dpi	*n*
**High Dose (Infected)**	600 µl Blood + 200 µl TrV Stock	5 x 10^7 copies	1, 3, 5, 7, 9, 12, 14, 16	96
**High Dose Control**	600 µl Blood + 200 µl TE Buffer	-		90
**Low Dose (Infected)**	600 µl Blood + 200 µl TrV Stock	2.25 x 10^6 copies	1, 3, 5, 7, 9, 12	88
**Low Dose Control**	600 µl Blood + 200 µl TE Buffer	-		75

Abbreviations: **dpi**, days post-inoculation; ***n***, sample size; **TE**, Tris-EDTA buffer (10 mM Tris-HCl, 1 mM EDTA, pH 7.4); **TrV**, *Triatoma virus*.

Table S4. Statistical evaluation of viral replication or purification efficiency across different experimental groups. Top: Global multi-group comparison executed via one-way Analysis of Variance (ANOVA) ($F_{3, 27} = 6.23$, $p = 0.0024$). Bottom: Post-hoc all-pairwise comparisons evaluated via Tukey's Honestly Significant Difference (HSD) test ($\alpha = 0.05$). Adjusted p-values are detailed alongside their statistical significance levels (ns: non-significant, *: $p < 0.05$; ** $p < 0.01$ ***: $p < 0.001$).

One-way ANOVA summary for viral RNA yield ($\log_{10}$ copies) across extraction protocols

	Df	Sum Sq	Mean Sq	*F* value	Pr(>F)
Method	3	24.93	8.31	6.23	0.0024 **
Residuals	27	35.99	1.33		
Total	30	60.92			

Tukey's Honestly Significant Difference (HSD) test (BH Adjusted)

group1	group2	Estimate	Confidence low	Confidence high	*p* adjusted	*p* signifies
Martí	Susevich	0.531286	-0.96746	2.030033	0.767	ns
Martí	EGM	0.973116	-0.43992	2.386149	0.258	ns
Martí	Microcentrifuge	-2.22974	-4.30967	-0.14981	0.0322	*
Susevich	EGM	0.44183	-1.05692	1.940578	0.851	ns
Susevich	Microcentrifuge	-2.76103	-4.90011	-0.62194	0.00771	**
EGM	Microcentrifuge	-3.20286	-5.28279	-1.12293	0.00135	**

Table S5 Pairwise statistical comparison of viral load kinetics between High-dose and Low-dose experimental cohorts. Pairwise comparisons of target quantification between consecutive sampling days post-infection (dpi) stratified by viral dose group. Unadjusted raw p-values were determined using Student's two-tailed t-test. Multiple testing correction across all pairwise comparisons were performed using the Benjamini-Hochberg (BH) procedure to control the false discovery rate (FDR). Asterisks indicate adjusted p-values considered statistically (ns: non-significant, $p >= 0.05$; *: $p < 0.05$; **: $p < 0.01$).

Dose Group	Comparison (dpi)	Comparison (dpi)	Statistical Test	Raw *p*-value	Adjusted *p*-value (BH)	Significance
Low	Day 1	Day 3	t-test	0.71	0.71	ns
**Low**	**Day 3**	**Day 7**	**t-test**	**0.011**	**0.022**	*
**Low**	**Day 7**	**Day 9**	**t-test**	**0.008**	**0.022**	*
Low	Day 9	Day 12	t-test	0.687	0.71	ns
High	Day 1	Day 3	t-test	0.025	0.071	ns
High	Day 3	Day 5	t-test	0.353	0.412	ns
High	Day 5	Day 7	t-test	0.938	0.938	ns
High	Day 7	Day 9	t-test	0.030	0.071	ns
**High**	**Day 9**	**Day 12**	**t-test**	**0.002**	**0.016**	*
High	Day 12	Day 14	t-test	0.123	0.181	ns
High	Day 14	Day 16	t-test	0.129	0.181	ns

Table S6. Summary statistics of Log-Rank (Mantel-Cox) test for survival comparisons. Summary of Kaplan-Meier survival analysis in *Rhodnius prolixus* challenged with Low and High doses of Triatoma virus (TrV) versus uninfected controls, as well as a direct dose-response comparison between infected groups (Low TrV vs. High TrV). Analytical sacrifices performed for kinetics monitoring (qPCR) were incorporated as right-censored observations (Status = 0) on the respective sampling timepoints. Statistical significance between curves was evaluated using the Log-Rank (Mantel-Cox) test ($\alpha = 0.05$).

Dose	Group	N	Observed deaths	Expected deaths	Chisq (χ^2)	*df*	*p* Value
Low	Control	75	5	6.53			
	TrV	88	10	8.47	0.65	1	0.42
High	Control	90	4	8.98			
	TrV	96	14	9.02	5.66	1	0.0174
Low		88	10	12.33			
High	TrV	96	14	11.67	0.96	1	0.32705

Abbreviations: **N**, initial cohort size reconstructed accounting for destructive sampling; **Observed**, cumulative recorded mortality events (Status = 1); **Expected**, expected number of mortality events under the null hypothesis; χ^2, Chi-square test statistic; *df*, degrees of freedom; ***p*-value**, nominal log-rank test probability value. Statistical significance was established at $p < 0.05$.
